# Parallelized gene cluster editing illuminates mechanisms of epoxyketone proteasome inhibitor biosynthesis

**DOI:** 10.1093/nar/gkad009

**Published:** 2023-01-31

**Authors:** Chuan Huang, Daniel Zabala, Emmanuel L C de los Santos, Lijiang Song, Christophe Corre, Lona M Alkhalaf, Gregory L Challis

**Affiliations:** Department of Chemistry, University of Warwick, Coventry CV4 7AL, UK; Warwick Integrative Synthetic Biology Centre, University of Warwick, Coventry CV4 7AL, UK; Biomedicine Discovery Institute, Department of Biochemistry and Molecular Biology, Monash University, Clayton, Victoria 3800, Australia; ARC Centre of Excellence for Innovations in Peptide and Protein Science, Monash University, Clayton, Victoria 3800, Australia; Department of Chemistry, University of Warwick, Coventry CV4 7AL, UK; Department of Chemistry, University of Warwick, Coventry CV4 7AL, UK; Warwick Integrative Synthetic Biology Centre, University of Warwick, Coventry CV4 7AL, UK; School of Life Sciences, University of Warwick, Coventry CV4 7AL, UK; Department of Chemistry, University of Warwick, Coventry CV4 7AL, UK; Department of Chemistry, University of Warwick, Coventry CV4 7AL, UK; Warwick Integrative Synthetic Biology Centre, University of Warwick, Coventry CV4 7AL, UK; School of Life Sciences, University of Warwick, Coventry CV4 7AL, UK; Department of Chemistry, University of Warwick, Coventry CV4 7AL, UK; Department of Chemistry, University of Warwick, Coventry CV4 7AL, UK; Warwick Integrative Synthetic Biology Centre, University of Warwick, Coventry CV4 7AL, UK; Biomedicine Discovery Institute, Department of Biochemistry and Molecular Biology, Monash University, Clayton, Victoria 3800, Australia; ARC Centre of Excellence for Innovations in Peptide and Protein Science, Monash University, Clayton, Victoria 3800, Australia

## Abstract

Advances in DNA sequencing technology and bioinformatics have revealed the enormous potential of microbes to produce structurally complex specialized metabolites with diverse uses in medicine and agriculture. However, these molecules typically require structural modification to optimize them for application, which can be difficult using synthetic chemistry. Bioengineering offers a complementary approach to structural modification but is often hampered by genetic intractability and requires a thorough understanding of biosynthetic gene function. Expression of specialized metabolite biosynthetic gene clusters (BGCs) in heterologous hosts can surmount these problems. However, current approaches to BGC cloning and manipulation are inefficient, lack fidelity, and can be prohibitively expensive. Here, we report a yeast-based platform that exploits transformation-associated recombination (TAR) for high efficiency capture and parallelized manipulation of BGCs. As a proof of concept, we clone, heterologously express and genetically analyze BGCs for the structurally related nonribosomal peptides eponemycin and TMC-86A, clarifying remaining ambiguities in the biosynthesis of these important proteasome inhibitors. Our results show that the eponemycin BGC also directs the production of TMC-86A and reveal contrasting mechanisms for initiating the assembly of these two metabolites. Moreover, our data shed light on the mechanisms for biosynthesis and incorporation of 4,5-dehydro-l-leucine (dhL), an unusual nonproteinogenic amino acid incorporated into both TMC-86A and eponemycin.

## INTRODUCTION

Rapid progress in DNA sequencing and bioinformatics has revealed opportunities as well as challenges for studying the biosynthesis of structurally complex specialized metabolites in microorganisms (1,2). To overcome some of the challenges, such as genetic intractability, complicated systems for regulation of gene expression, and complex culture conditions that are difficult to reproduce, expression of biosynthetic gene clusters (BGCs) in well-characterized heterologous hosts is often employed (3,4). This requires effective methods for cloning and genetic manipulation of BGCs.

Several approaches for cloning whole BGCs have been developed, including direct capture from genomic DNA (e.g. using transformation-associated recombination (TAR), RecET recombineering, Cas9-mediated targeting of chromosome segments and *in vitro* λ packaging and ligation) (5–8) and BGC assembly (e.g. DNA assembler and Direct Pathway Cloning) (9,10). Among these methods, TAR cloning is widely used for direct capture of chromosomal fragments from diverse species into a circular artificial chromosome that can propagate, segregate, and be selected for in yeast (11). To facilitate the heterologous expression of directly cloned bacterial BGCs in *Streptomyces* hosts, the Moore lab developed a yeast-*Escherichia coli-Streptomyces* shuttle vector, pCAP01 which can be employed to capture a specific BGC via homologous recombination with two ∼1 kb flanking sequences (5). One disadvantage of this method is the high background level of plasmid recircularization via non-homologous end joining. To solve this problem and simplify vector construction, pCAP03 was developed. This utilizes two ∼50 bp BGC flanking sequences inserted between a counter-selectable marker (*URA3*) and its promoter (*pADH1*) (12). However, a potential drawback of this method is that the short flanking sequences may lower the homologous recombination efficiency for some BGCs.

In-frame gene deletion is a powerful approach for elucidating the biosynthetic functions of proteins encoded by BGCs (13–18). However, most methods for constructing such deletions involve several time-consuming and labor-intensive steps, necessitating the serial construction and analysis of numerous mutants to investigate the function of all proteins encoded by a typical BGC. The development of parallelizable methods for construction and analysis of gene deletions would significantly accelerate our understanding of specialized metabolite biosynthesis.

Early approaches to the construction of in-frame gene deletions involved two sequential homologous recombination events in the native metabolite producer using marker-assisted selection and screening (19), which is a slow and laborious process. More recently, tools for efficient recombination in *E. coli* (e.g. λ-Red-mediated recombination) have streamlined the process of creating such mutants. Using a PCR-generated resistance cassette, genes in cosmids containing partial BGCs can be replaced in a single step (20). The engineered cosmids can be used to replace the corresponding genes on the chromosomes of native metabolite producers via double homologous recombination and the resistance gene can be excised from the resulting mutants to create in-frame deletions containing a ‘scar’ sequence (20). In addition to being difficult to parallelize, these methods are only applicable to genetically tractable native producers, or cloned BGCs that have already been incorporated into a heterologous host.

The ability to routinely clone complete BGCs enables the creation of in-frame deletions prior to expression in a heterologous host, which is easier to parallelize. TAR in yeast has been combined with selectable markers to swap targeted regions (5,21). Moreover, double-strand break (DSB)-stimulated recombination, initiated using naturally occurring or artificially introduced unique restriction sites, enables various modifications of cloned BGCs, including promoter engineering, gene deletion and targeted mutation (22,23). Site-specific DSBs can also be created *in vivo* or *in vitro* using the programmable CRISPR/Cas9 endonuclease editing system. This enables multiplexed creation of unmarked gene deletions (CReasPy-Cloning), promoter engineering (mCRISTAR, miCASTAR) and site-directed mutagenesis in clones containing whole BGCs (24–28). However, CRISPR/Cas9-based methods are often hampered by off-target effects that yield unpredictable DSBs and/or require tedious processes for multiplexing, such as the manufacture and introduction of customized gRNA expression plasmids for each *in vivo* modification, or optimization of multiple parameters for efficient digestion at each site *in vitro*. Direct assembly of overlapping DNA fragments in yeast offers an alternative approach for multiplexed BGC manipulation. However, this method becomes prohibitively expensive for large BGCs and unintended mutations resulting from errors in DNA synthesis must be excluded prior to assembly (29). Thus, despite considerable promise, yeast has yet to be developed into a widely applicable, operationally simple, and cost-effective platform for cloning and parallelized editing of complete BGCs.

**Figure 1.**
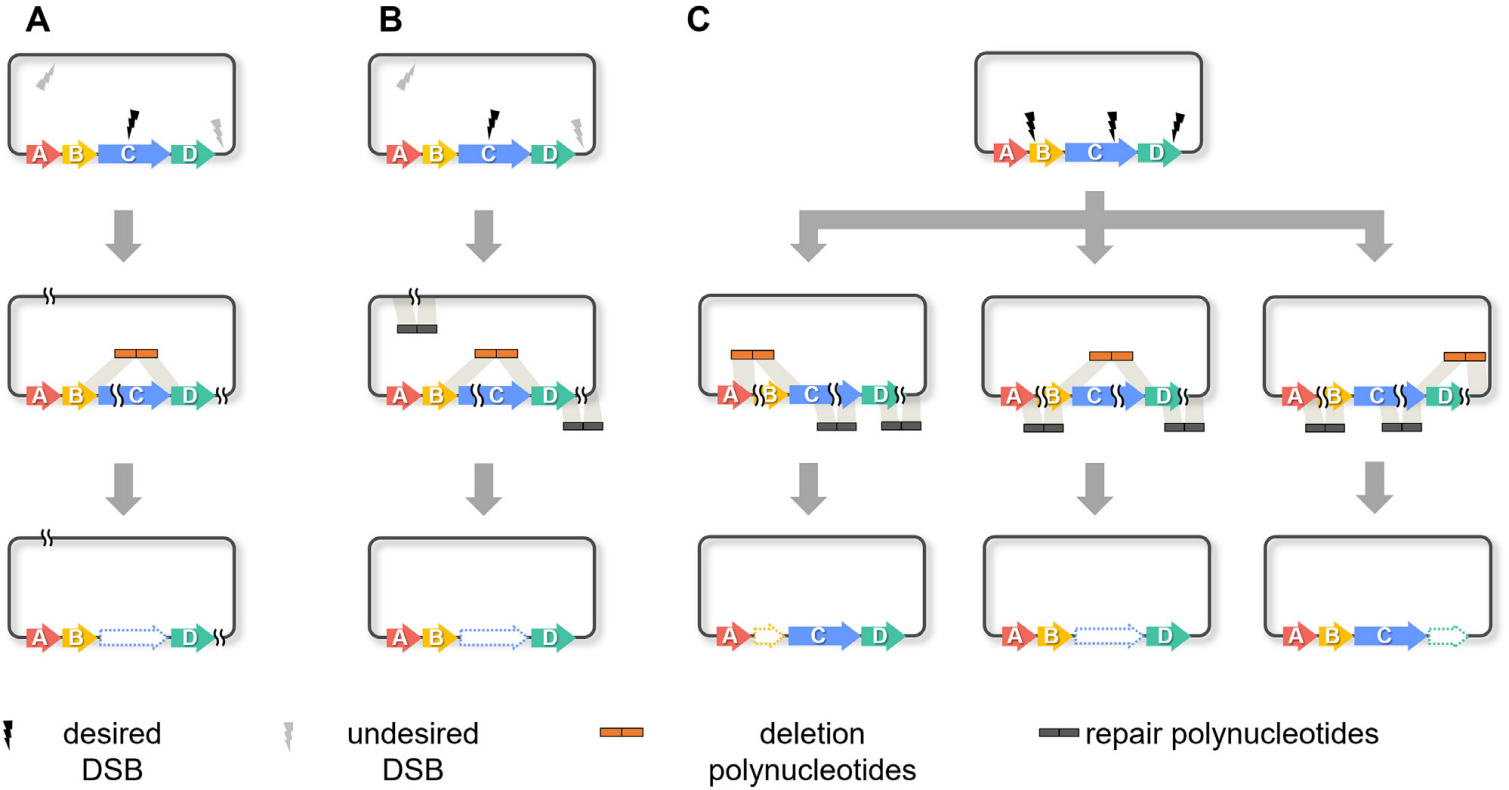
Scheme highlighting the benefits of the approach described here over other methods for yeast-mediated editing of cloned BGCs. (**A**) Linearization of the vector using a unique restriction site in the target gene, or CRISPR-Cas9, enables an in-frame deletion to be created by co-transformation with a deletion polynucleotide (23). However, in practice most genes in a BGC do not contain a unique restriction site and CRISPR-Cas9 can be hampered by off-target effects leading to additional undesired DSBs at unpredictable locations. These problems limit the general applicability of this approach. (**B**) For genes lacking a unique restriction site, a DSB is created in the target gene along with a handful of undesired DSBs at other sites; however, their location is predictable. Thus, co-transformation of yeast with the digested vector, a deletion polynucleotide, and several repair polynucleotides enables an in-frame deletion to be introduced into every gene in the BGC. (**C**) The same restriction site is often present in several different genes, enabling several deletion mutants to be created from a vector digested with a single endonuclease by multiplexed reassembly.

Here, we describe the development of a yeast-based platform for efficient capture and genetic manipulation of complete BGCs. Key features of this platform include: (i) the use of commercially available restriction endonucleases to create a predictable set of DSBs in vectors containing captured BGCs; (ii) efficient TAR-mediated reassembly of digested vectors into derivatives containing in-frame deletions in target genes using different combinations of repair and deletion polynucleotides and (iii) modest amounts of PCR screening (typically <20 colonies) to identify clones with the desired genotype (Figure 1A and B). The multiplexability of the restriction digestion-reassembly cascade (Figure 1C), coupled with the operational simplicity of the overall process, greatly facilitates parallelized construction of BGC variants.

**Figure 2.**
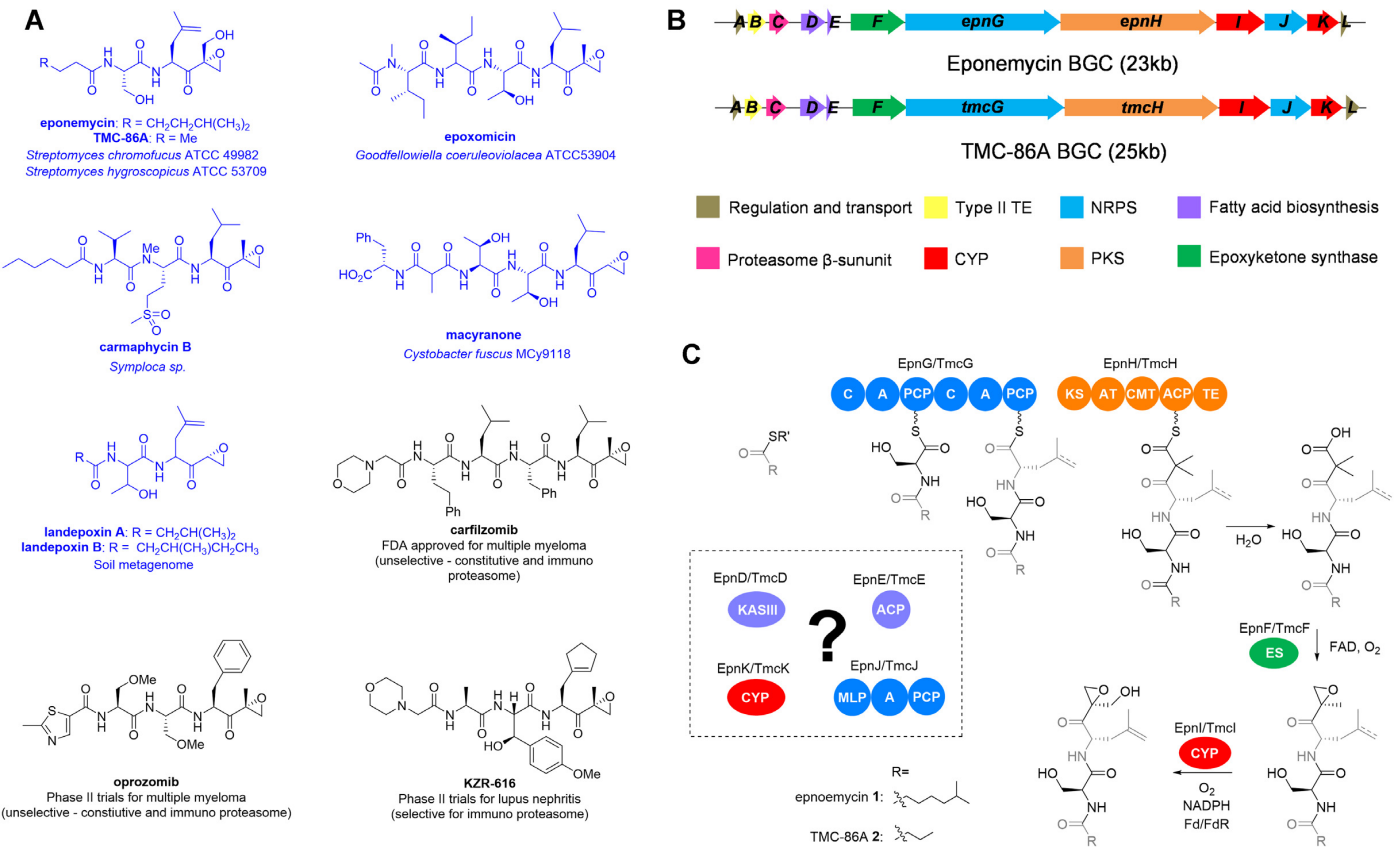
Structures, origins, and therapeutic applications of epoxyketone proteasome inhibitors, and comparison of the biosynthetic gene clusters and proposed biosynthetic pathways for eponemycin and TMC-86A. (**A**) Structures and sources of natural epoxyketones (in blue), and structures and applications of synthetic epoxyketones (in black) inspired by the natural products. (**B**) Comparison of the *S*.*hygroscopicus* eponemycin and *S. chromofuscus* TMC-86A BGCs. (**C**) Previously proposed biosynthetic pathways for eponemycin **1** and TMC-86A **2** in *S*.*hygroscopicus* and *S*.*chromofuscus* respectively. The variable *N*-acyl group and conserved dhL residue in eponemycin and TMC-86A, which are assembled via uncharacterized mechanisms, are shown in grey. The dashed 4,5-double bond in the dhL residue indicates that the timing of its introduction is unknown. The dashed box contains putative biosynthetic enzymes with experimentally unvalidated functions encoded by the eponemycin and TMC-86A BGCs. R′ = ACP or CoA.

As a proof of concept, we have applied this platform to BGCs directing the biosynthesis of eponemycin (1) and TMC-86A (2), two members of a group of epoxyketone proteasome inhibitors that inspired the development of the multiple myeloma drug Carfilzomib and the orally bioavailable derivative Oprozomib, in addition to selective inhibitors of the immunoproteasome for the treatment of autoimmune diseases (Figure 2A) (30,31). Epoxyketones have been isolated from various microbes, including Actinobacteria, Cyanobacteria, Myxobacteria and environmental microbiomes (Figure 2A) (32–37). The epoxyketone moiety specifically targets the catalytic threonine residue in the β-subunit of the proteasome (32,33,38,39). Very similar BGCs for eponemycin (1) and TMC-86A (2), which bear a close structural relationship, have been identified in *Streptomyces hygroscopicus* ATCC 53709 and *Streptomyces chromofuscus* ATCC 49982 respectively (Figure 2A and B) (40,41). Previous *in vitro* reconstitution experiments showed that the epoxyketone warhead of TMC-86A and eponemycin is assembled from an α-dimethyl-β-keto acid biosynthetic intermediate by the unique flavin-dependent decarboxylase-desaturase-monooxygenase TmcF/EpnF and undergoes subsequent hydroxylation by the cytochrome P450 (CYP) TmcI/EpnI (40).

The biosynthesis of TMC-86A is proposed to commence with the condensation of a butanoyl thioester with l-Ser and l-Leu, catalyzed by the nonribosomal peptide synthetase (NRPS) TmcG (40). The resulting *N*-acyl-dipeptidyl thioester is then elongated with a malonyl extender unit and dimethylated by the polyketide synthase (PKS) TmcH (Figure 2C), prior to thioesterase domain-mediated hydrolysis from the PKS to produce the α-dimethyl-β-keto acid intermediate (40). Eponemycin is proposed to be biosynthesized via an analogous pathway (Figure 2C), except that a 6-methylheptanoyl thioester is used as the starter unit (42,43). However, the mechanisms responsible for incorporation of different starter units and the common dhL residue into TMC-86A and eponemycin remain unclear, notwithstanding previous efforts to genetically characterize the eponemycin BGC (42,43). Our results demonstrate that both BGCs direct the biosynthesis of TMC-86A, using the ketoacyl-ACP synthase III (KASIII) homologue TmcD/EpnD and the acyl carrier protein (ACP) TmcE/EpnE to generate the butanoyl thioester starter unit. Eponemycin and related congeners are shunt metabolites that appear to result from hijacking of alternative starter units from the primary metabolic fatty acid synthase (FAS). Moreover, they show that desaturation of the l-Leu side chain precedes eponemycin assembly and involves the CYP EpnK and the NRPS EpnJ.

## MATERIALS AND METHODS

### Bacterial strains, plasmids and culture conditions

The strains and plasmids used in this study are listed in Supplementary Table S1. *E*.*coli* Top10 (Invitrogen), used for routine cloning, was grown in Luria-Bertani medium (tryptone 1%, yeast extract 0.5%, NaCl 1%) at 37°C with appropriate antibiotic selection when necessary (ampicillin 100 μg/ml, apramycin 50 μg/ml, kanamycin 25 μg/ml or chloramphenicol 25 μg/ml).

*S*.*hygroscopicus* ATCC53709, *S*.*chromofuscus* ATCC49982, and *Streptomyces albus* J1074 were grown on either TSB medium (Becton Dickinson) or SFM medium (19) at 30°C for routine cultivation. For epoxyketone production, 20 μl spore suspension was inoculated on 25 ml R5 agar medium (19) and incubated at 30°C for 7 days. For the dhL feeding experiment, substrate was dissolved in 1 ml water and overlaid on the agar plates after 48 h from culture initiation (10 mM final concentration).

*Saccharomyces cerevisiae* VL6 − 48N was grown on YPD medium (glucose 2%, yeast extract 1%, peptone 2%) supplemented with 100 mg/l adenine at 30°C. For TAR cloning, tryptophan-deficient medium (sorbitol 18.2%, glucose 2.2%, yeast nitrogen base without amino acid and ammonium sulfate 0.17%, yeast synthetic drop-out medium supplements without tryptophan 0.19%, ammonium sulfate 0.5% and agar 2%) was used with 5-Fluoroorotic acid (1 mg/ml) when necessary (12).

### Construction of plasmids

DNA isolation and manipulation was carried out according to standard methods for *E*.*coli* and *Streptomyces* (19,44). To generate the *pADH1*-*URA3* cassette for pCAP1000, *pADH1* and *URA3* genes were amplified from pCAP03 separately and joined using overlap PCR (see primer list in Supplementary Table S2). For pCAP1000 linearization before yeast transformation, BamHI, PmeI and NdeI restriction sites were introduced between *pADH1* and *URA3*. The *pADH1*-*URA3* cassette was sub-cloned into the pCR™2.1 vector for verification. After verification by DNA sequencing, the *pADH1*-*URA3* cassette was amplified (see Primers List in Supplementary Table S2) and cloned into SpeI and XhoI-digested pCAP01 using Gibson Assembly to generate pCAP1000.

For pCAP03-based vectors, homologous arms were synthesized and cloned into pCAP03 by GENEWIZ. For pCAP1000 and pCAP01-based vectors, homologous arms for each clusters were amplified from genomic DNA of each wild type strain using Phusion^®^ High-Fidelity DNA polymerase (New England Biolabs), with ∼15 bp primer-introduced overhangs for Gibson Assembly/GeneArt™ Seamless Cloning and Assembly. The counter-selectable cassettes were amplified from pCR-ADHURA3 or pCAP1000 using Phusion^®^ High-Fidelity DNA polymerase, with ∼15 bp primer-introduced overhangs for Gibson Assembly/GeneArt™ Seamless Cloning and Assembly. All paired homologous arms were cloned into SpeI/XhoI-digested pCAP01 (without counter-selectable cassettes) and SpeI/XhoI-digested pCAP1000 (together with counter-selectable cassettes) to create the following plasmids: pCAP01epn and pCAP1000epn for epnBGC capture; pCAP01tmc and pCAP1000tmc for tmcBGC capture. The integrity of all plasmids was confirmed by restriction digestion and sequencing.

For genetic complementation experiments, target genes were amplified from genomic DNA using Phusion DNA polymerase and cloned into pOSV556t via HindIII/StuI restriction sites to generate pOSV-*epnD*, pOSV-*epnE*, pOSV-*epnDE*, pOSV-*tmcD*, pOSV-*epnI* and pOSV-*epnK*. All constructs were verified by restriction digestion and sequencing.

### Yeast transformation and *Streptomyces*/*E*. *coli* conjugation

To create appropriate genomic fragments for TAR cloning of BGCs, genomic DNA isolated from the eponemycin and TMC-86A producing organisms was digested with suitable restriction enzymes (PstI for the epnonemycin BGC, and NdeI and XhoI for the TMC86A BGC), and purified by ethanol precipitation. 2–4 μg of digested genomic DNA were used for yeast spheroplast transformation, together with 0.1–0.2 μg of the appropriate PmeI-linearized capture vector. For gene editing, plasmids were digested with appropriate restriction enzymes that cut within or adjacent to target genes and purified by ethanol precipitation. 0.1–0.2 μg of digested plasmid and 0.05–0.1 μg of the requisite repair and deletion polynucleotides were used to co-transform yeast spheroplasts. Yeast spheroplast preparation and transformation were carried out using a previously reported protocol (11). After spheroplast transformation, yeast colonies were picked and screened by PCR using a previously reported protocol with primers designed to amplify fragments from the middle and each end of the target BGC (11). Plasmids were isolated from PCR-positive yeast colonies and used to transform *E. coli* Top10. After verification by restriction digestion, plasmids harboring target BGCs or their derivatives were introduced into *S*.*albus* J1074 by triparental mating from *E*.*coli* ET12567, via the helper strain *E*.*coli* ET12567/pUB307, using a standard protocol (45). Kanamycin resistance was used for exconjugant screening.

Genetic complementation plasmids were introduced into *S*.*albus*/epnBGC-Δ*epnD* (pOSV-*epnD*), *S*.*albus*/epnBGC-Δ*epnE* (pOSV-*epnE*), *S*.*albus*/epnBGC-Δ*epnDE* (pOSV-*epnDE*), *S*.*albus*/tmcBGC-Δ*tmcD* (pOSV-*tmcD* and pOSV-*epnD*), *S*.*albus*/epnBGC-Δ*epnI* (pOSV-*epnI*), *S*.*albus*/epnBGC-Δ*epnK* (pOSV-*epnK*) and *S*.*albus*/epnBGC-Δ*epnJ* (pOSV-*epnJ*) via conjugal transfer from *E. coli* ET12567/pUZ8002. Hygromycin was used for transconjugant selection.

### Growth of *Streptomyces* strains and analysis of epoxyketone production

After 7 days incubation, the agar was cut into small chunks and 15 ml of ethyl acetate was added to each plate. After 2 h, the organic extracts were decanted and concentrated by rotary evaporation. The residues were separately dissolved in 1 ml of methanol, and 2 μl of each sample were analyzed by UHPLC-ESI-Q-TOF-MS/MS on a Bruker MaXis II mass spectrometer (or Bruker MaXis Impact mass spectrometer) coupled to a Dionex UltiMate 3000 UHPLC fitted with an Agilent Zorbax Eclipse Plus C18 column (100 × 2.1 mm, 1.8 μm). Using a flow rate of 0.2 ml/min, the column was eluted with a combination of water and acetonitrile as follows: 5% (v/v) acetonitrile for 5 min, 5−100% (v/v) acetonitrile over 21 min, 100% (v/v) acetonitrile for 3 min, 100−5% (v/v) acetonitrile over 2 min and 5% (v/v) acetonitrile for 3 min.

## RESULTS

### Cloning and heterologous expression of the eponemycin and TMC-86A BGCs

To capture the eponemycin and TMC-86A BGCs, we initially constructed pCAP03-based vectors containing 50 bp flanking sequences (pCAP03epnBGC and pCAP03tmcBGC, respectively). However, no clones were obtained for either cluster using these vectors, despite several attempts (Supplementary Table S3). Reasoning that low homologous recombination efficiency, due to the short flanking sequences, was the cause of this failure, we next attempted to capture the two clusters using pCAP01 containing 1 kb flanking sequences. While we succeeded in cloning the eponemycin BGC using this approach, a high level of plasmid recircularization was observed, and we still failed to clone the TMC-86A BGC (Supplementary Table S3).

**Figure 3.**
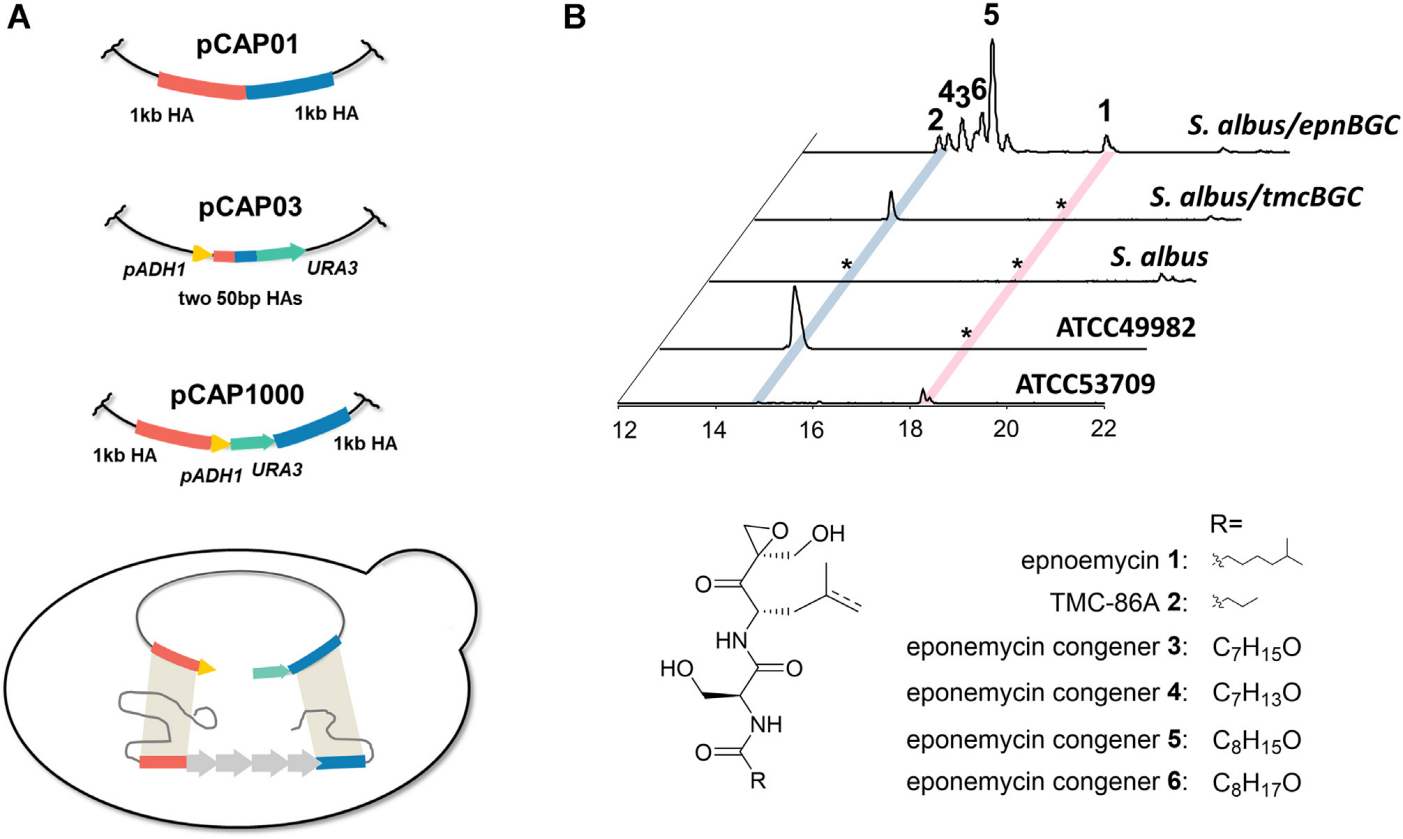
Cloning and heterologous expression of the eponemycin and TMC-86A BGCs using pCAP1000. (**A**) Comparison of homologous arms (HAs) and counter-selectable markers incorporated into pCAP01, pCAP03 and pCAP1000. The two HAs are shown in red and blue, respectively. The pADH1 promoter is shown in yellow and URA3 is shown in green. The combination of 1kb HAs and a counter-selectable marker in pCAP1000 improves the efficiency of BGC capture via double homologous recombination, which is illustrated schematically below the vector comparison. (**B**) Extracted ion chromatograms at *m/z* = 421.2309 ± 0.002, 365.1683 ± 0.002, 437.2258 ± 0.002, 435.2102 ± 0.002, 451.2415 ± 0.002 and 449.2258 ± 0.002, corresponding to [M + Na]+ for eponemycin **1**, TMC-86A **2**, and eponemycin congeners **3**, **4**, **5** and **6**, respectively, from UHPLC-ESI-Q-TOF-MS analysis of culture extracts of *S. albus* with and without pCAP1000epnBGC and pCAP1000tmcBGC integrated into its chromosome, the eponemycin producer *S. hygrosopicus* ATCC53709, and the TMC-86A producer *S. chromofuscus* ATCC49982.

To surmount these problems, we sought to use 1 kb flanking sequences in conjunction with the *URA3* counter selectable marker. However, this was not possible in pCAP03 because the flanking sequences are inserted between the *URA3* gene and its promoter (*pADH1*) (12,21,46,47), which limits their combined length to 130 bp. We therefore cloned a modified *pADH1*-*URA3* cassette into pCAP01 to create pCAP1000 (Figure 3A). The 1kb flanking sequences initially inserted in pCAP01 were then cloned into pCAP1000 either side of the *pADH1*-*URA3* cassette, generating pCAP1000epn and pCAP1000tmc, respectively. Using these vectors, we succeeded in cloning both clusters and a low background of plasmid recircularization was observed (four out of six clones correct using pCAP1000epn and all four clones correct using pCAP1000tmc; see Supplementary Table S3). The inserts of the resulting plasmids, pCAP1000epnBGC and pCAP1000tmcBGC-initial, were validated using restriction enzyme digests (Supplementary Figure S1).

The plasmids were integrated into the chromosome of *S*.*albus* J1074 via triparental conjugation (48), and the resulting strains were cultivated for 7 days at 30°C on R5 agar medium. UHPLC-ESI-Q-TOF-MS analysis of ethyl acetate culture extracts identified six metabolites in the strain containing pCAP1000epnBGC that were absent from wild type *S. albus*. Two of these were identified as eponemycin **1** and TMC-86A **2**, based on molecular formulae deduced from high resolution mass spectra and retention time comparisons with authentic standards produced by *S. hygroscopicus* ATCC 53709 and *S. chromofuscus* ATCC 49982, respectively (Figure 3B and Supplementary Figure S3). The remaining four metabolites yielded [M+H]^+^ ions with *m/z* = 413.2286 (calculated for C_20_H_33_N_2_O_7_^+^: 413.2282), *m/z* = 415.2444 (calculated for C_20_H_35_N_2_O_7_^+^: 415.2439), *m/z* = 427.2440 (calculated for C_21_H_35_N_2_O_7_^+^: 427.2439) and *m/z* = 429.2599 (calculated for C_21_H_37_N_2_O_7_^+^: 429.2595) and were proposed to be eponemycin congeners **3**, **4**, **5** and **6** with alterations to the N-acyl group (Supplementary Figure S2). MS/MS analyses were consistent with these structural assignments (Supplementary Figure S3).

In contrast, no new metabolites were produced by *S. albus* containing pCAP1000tmcBGC-initial. Close inspection of the insert captured in this plasmid indicated that the promoter region upstream of *tmcA* (which encodes a putative LuxR-like transcriptional activator) is truncated, likely affecting the expression of this gene (40,43). Reasoning that TmcA may function as a transcriptional activator of the downstream biosynthetic genes, we amplified 995 bp from the intergenic region upstream of *tmcA* flanked by 50 bp arms with homology to the 5′ end of the insert and the adjacent vector backbone. A DSB upstream of the *tmcA* start codon was generated using a unique XhoI restriction site and yeast was co-transformed with the linearized plasmid and the PCR product, leading to incorporation of the 995 bp intergenic region into pCAP1000tmcBGC-initial. The resulting construct (named pCAP1000tmcBGC) was integrated into the chromosome of *S. albus* and, after 7 days cultivation at 30°C on R5 agar medium, UHPLC-ESI-Q-TOF-MS analysis of ethyl acetate extracts showed that this strain produces TMC-86A **2** (Figure 3B). Interestingly, neither eponemycin **1**, nor its congeners **3, 4, 5** and **6** were detected in the extracts (Figure 3B).

### Parallelized construction of in-frame deletions in *epnD*, *epnE*, *epnI*, *epnJ* and *epnK*

Previous attempts to elucidate the roles in eponemycin biosynthesis played by *epnD*, *epnE*, *epnI*, *epnJ* and *epnK* via construction and analysis of the corresponding in-frame deletion mutants failed to provide useful insights (43). During resequencing of the eponemycin BGC cloned into pCAP1000, we identified several errors in the originally reported sequence (41). In particular, cytosine and guanine residues are missing from the originally reported sequences of *epnD* and *epnK*, respectively. These errors resulted in frame shifts in the originally reported *epnD* and *epnK* sequences that eliminated a stop codon 27 nucleotides downstream of the originally assigned start codon and introduce a stop codon 11 nucleotides upstream of the true stop codon, respectively (Supplementary Figure S4). Thus, *epnD* is 27 bp shorter and *epnK* is 15 bp longer than originally assigned. These errors likely contributed to the failure of the previous genetic studies to establish clear roles for several of the eponemycin biosynthetic genes.

**Figure 4.**
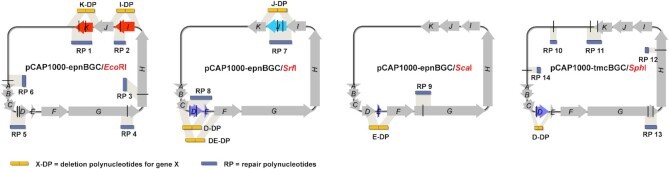
Scheme illustrating parallelized construction of in-frame deletions in the eponemycin and TMC-86A BGCs using homology-directed DSB repair in yeast. DSBs are generated by restriction enzyme digestion in vitro. Using different combinations of repair polynucleotides (RP) and deletion polynucleotides (DP), the fragmented plasmids can be reassembled in yeast to create a series of in-frame deletion mutants.

To reevaluate the roles of *epnD*, *epnE*, *epnI*, *epnJ* and *epnK* in eponemycin biosynthesis we designed a yeast-based method for parallelized construction of in-frame deletions in these five genes. Three different restriction enzymes were employed to create one or two DSBs in each gene: EcoRI for *epnI* and *epnK*, SrfI for *epnJ* and *epnD*, and ScaI for *epnE* (Figure 4). These enzymes introduced DSBs at up to six other locations in pCAP1000epnBGC, resulting in a total of eight, four and two fragments from the EcoRI, SrfI and ScaI digests, respectively. 1 kb repair polynucleotides that symmetrically span the DSB (or pair of DSBs if they are within 1 kb of each other) were amplified from pCAP1000epnBGC for each off target cut site and 1 kb deletion polynucleotides for each target gene were assembled by overlap PCR using two 500 bp amplimers from the 5′ and 3′ flanking regions. The fragments from each restriction digest were mixed with the appropriate combination of repair and deletion polynucleotides and used to transform yeast. 60–100% of transformants resulting from the mixtures containing the SrfI and ScaI restriction fragments harboured reassembled plasmids with the desired genotype (Supplementary Table S4). Reassembly efficiency was reduced when the EcoRI restriction fragments were used (13–15% positives—see Supplementary Table S4), but only a modest amount of screening was needed to identify a correct clone. The resulting plasmids, named pCAP1000epnBGC-△*epnX* (where X is the identifying letter for each gene), contained 1188, 984, 186, 1080 and 1509 bp in-frame deletions in *epnI, epnD, epnE*, *epnK* and *epnJ*, respectively. The genotypes of these plasmids were confirmed by restriction digestion and sequencing, and no adventitious mutations were detected (Supplementary Figure S1). Each plasmid was separately transferred into *S. albus* via conjugation from *E. coli*.

To further confirm the fidelity of plasmid reassembly, we initially investigated the production of eponemycin-related metabolites by the *epnI* mutant. The *epnI* gene encodes a protein with 89% identity to TmcI from *S. chromofuscus*, a CYP that catalyzes the final hydroxylation step in TMC-86A biosynthesis (Figure 2B) (40). UHPLC-ESI-Q-TOF-MS analysis of the *epnI* mutant showed that the production of both TMC-86A and eponemycin was abolished and two new products, with molecular formulae corresponding to deshydroxy-eponemycin **11** ([M+H]^+^*m/z* 383.2525, calculated for C_20_H_35_N_2_O_5_^+^: 383.2540) and deshydroxy-TMC-86A **12** ([M+H]^+^*m/z* 327.1914, calculated for C_16_H_27_N_2_O_5_^+^: 327.1914) were produced (Supplementary Figure S3). Moreover, production of the eponemycin congeners **3**, **4**, **5** and **6** was also abolished and new compounds giving rise to [M+H]^+^ ions with *m/z* = 397.2325 (calculated for C_20_H_33_N_2_O_6_^+^: 397.2333), 399.2484 (calculated for C_20_H_35_N_2_O_6_^+^: 399.2490), 411.2483 (calculated for C_21_H_35_N_2_O_6_^+^: 411.2490) and 413.2639 (calculated for C_21_H_37_N_2_O_6_^+^: 413.2646), consistent with molecular formulae for the deshydroxy derivatives of the congeners **13**, **14**, **15** and **16**, were observed (Supplementary Figure S2A). Comparison of the MS/MS spectra for **11**, **12**, **13**, **14**, **15** and **16**, with those for **1**, **2**, **3**, **4**, **5** and **6**, respectively, provided further evidence that the hydroxymethyl groups appended to the epoxyketone warhead in the latter are replaced by methyl groups in the former (Supplementary Figure S3). Introduction of pOSV-*epnI* (an integrative vector containing *epnI* under the control of the constitutive ermE* promoter) into the *epnI* mutant restored the production of eponemycin **1**, eponemycin congeners **3**–**6** and TMC-86A **2** (Supplementary Figure S2A). These data show that the function of all the eponemycin biosynthetic genes, except *epnI*, are unaffected in the pCAP1000epnBGC-△*epnI* construct, demonstrating that yeast-mediated plasmid reassembly proceeds with a very high degree of fidelity.

### EpnD and EpnE are required for assembly of the TMC-86A butanoyl group

**Figure 5.**
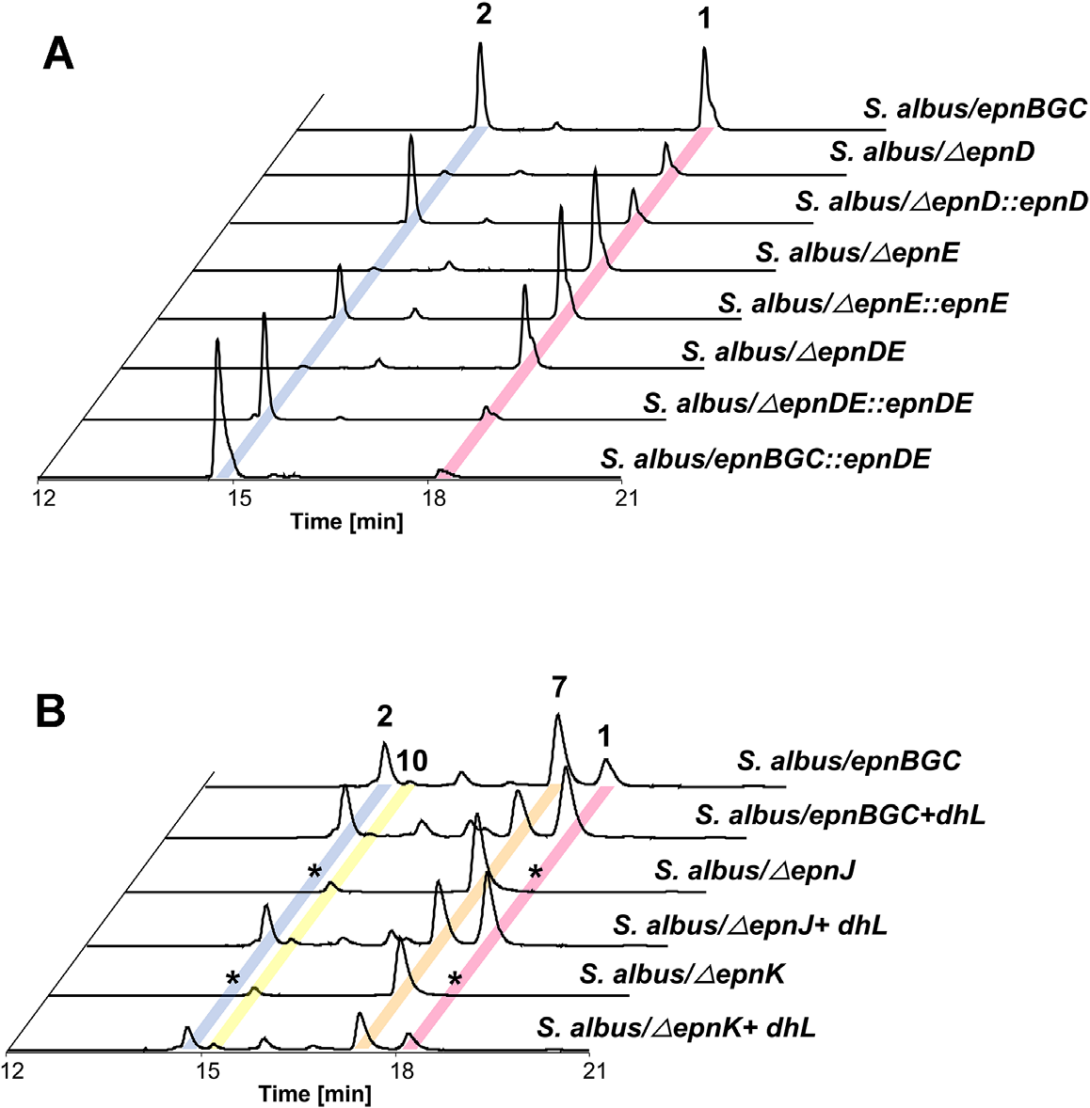
Extracted ion chromatograms (EICs) from UHPLC-ESI-Q-TOF-MS analyses of culture extracts from *S. albus* containing pCAP1000epnBGC and various engineered derivatives. (**A**) Comparison of EICs at *m/z* = 421.2309 ± 0.002 and 365.1683 ± 0.002, corresponding to the [M+Na]+ ions for eponemycin **1** and TMC-86A **2**, respectively, for *S. albus* containing pCAP1000epnBGC; Δ*epnD*, Δ*epnE* and Δ*epnDE* derivatives; Δ*epnD*, Δ*epnE* and Δ*epnDE* derivatives plus *epnD*, *epnE* and *epnDE* under the control of the constitutive ermE* promoter; and pCAP1000epnBGC plus *epnDE* under the control of the ermE* promoter. The corresponding EICs for eponemycin congeners **3**–**6** are in Supplementary Figure S2B. (**B**) EICs at *m/z* = 421.2309 ± 0.002, 365.1683 ± 0.002, 407.2516 ± 0.002, and 367.1840 ± 0.002, corresponding to the [M+Na]^+^ ions for eponemycin **1**, TMC-86A **2**, deshydroxy-dihydro-eponemycin congener **7**, and dihydroTMC-86A congener **10**, respectively, for *S. albus* containing pCAP1000epnBGC with and without added dhL, and Δ*epnK* and Δ*epnJ* derivatives with and without added dhL. The asterisks highlight the absence of peaks for **1** and **2** in the Δ*epnK* and Δ*epnJ* mutants. The corresponding EICs for eponemycin congeners **3**–**6**, dihdydro-eponemycin **8**, and a dihydro-eponemycin congener **9** are in Supplementary Figures S2D and S3.

To investigate the biosynthetic roles of the proteins encoded by *epnD* and *epnE*, which are KASIII and ACP homologs, respectively, the profile of metabolites produced by *S. albus* containing pCAP1000epnBGC, pCAP1000epnBGC-△*epnD* and pCAP1000epnBGC-△*epnE* were compared using UHPLC-ESI-Q-TOF-MS. While there was only a modest decrease in the production of eponemycin **1** and congeners **3**, **4**, **5** and **6** in the *epnD* mutant (∼70% of wild type), TMC-86A **2** production was strongly affected (Figure 5A and Supplementary Figure S2B). Introduction of pOSV-*epnD* into the Δ*epnD* mutant restored wild type levels of TMC-86A **2**, without affecting the production of eponemycin **1**, or congeners **3**, **4**, **5** and **6** (Figure 5A, Supplementary Figure S2B). In the *epnE* mutant, TMC-86A **2** production was also strongly affected, whereas eponemycin **1** and congeners **3**, **4**, **5** and **6** were produced at wild type levels (Figure 5A, Supplementary Figure S2B). Introduction of pOSV-*epnE* into the mutant restored TMC-86A **2** production to 50% of the wild-type level, without affecting the production of eponemycin **1**, or congeners **3**, **4**, **5** and **6** (Figure 5, Supplementary Figure S2B). These data indicate that EpnD and EpnE both play an important role in the biosynthesis of TMC-86A **2** but neither is required for the assembly of eponemycin **1** or its congeners.

To further confirm the key role played by EpnD and EpnE in TMC-86A **2** biosynthesis, we used our genetic manipulation platform to create a double mutant by deleting 1242 bp in pCAP1000epnBGC between the start codon of *epnD* and the stop codon of *epnE* (including the 18bp intergenic region; Figure 4 and Supplementary Table S4). The integrity of the resulting plasmid (pCAP1000epnBGC-△*epnDE*) was confirmed by restriction enzyme digestion and sequencing (Supplementary Figure S1), and it was introduced into *S. albus*. UHPLC-ESI-Q-TOF-MS analysis of ethyl acetate extracts demonstrated that TMC-86A **2** production is strongly suppressed in the resulting strain, whereas the levels of eponemycin **1** and congeners **3**, **4**, **5** and **6** are not significantly altered (Figure 5A, Supplementary Figure S2B). Introduction of pOSV-*epnDE* into the strain dramatically increased the production of TMC-86A **2** relative to eponemycin **1** and congeners **3**, **4**, **5** and **6** (Figure 5A, Supplementary Figure S2B). The more marked increase in titers of TMC-86A **2** relative to eponemycin **1** and congeners when *epnD* and *epnE* are co-expressed in the *epnDE* double mutant and epnBGC heterologous expression mutant compared to the complemented *epnD* and *epnE* individual mutants indicates that EpnD and EpnE cooperate closely in the assembly of the butanoyl starter unit for TMC-86A biosynthesis.

We also created a 984 bp in-frame deletion in *tmc*D (which encodes an EpnD orthologue) to investigate the function of this gene in the *S. chromofuscus* TMC-86A BGC. *Sph*I digestion of pCAP1000tmcBGC produced eight fragments, which were used with a *tmcD* deletion polynucleotide and four repair polynucleotides to co-transform yeast (Figure 4 and Supplementary Table S4). 1 out of 14 colonies screened contained the correctly reassembled plasmid (pCAP1000tmcBGC-△*tmcD*). The integrity of this plasmid was confirmed by restriction digestion and sequencing (Supplementary Figure S1), and it was transferred into *S. albus* by conjugation from *E. coli*. UHPLC-ESI-Q-TOF-MS analysis of culture extracts showed that TMC-86A **2** production is strongly suppressed in this strain and eponemycin **1** plus congeners **3**, **4**, **5** and **6** are produced (Supplementary Figure S2C). Introduction of either pOSV-*tmcD* or pOSV-*epnD* into this strain greatly increased the levels of TMC-86A **2** relative to eponemycin **1** and its congeners (Supplementary Figure S2C).

Collectively, these data show that the principal difference between the epoxyketone biosynthetic pathways in *S. hygroscopicus* and *S. chromofuscus* is the respective ability of EpnD/EpnE and TmcD/TmcE to supply the butanoyl starter unit to the NRPSs EpnG and TmcG. In the former, this process is relatively inefficient, allowing alternative starter units, presumably assembled by the primary metabolic FAS, to be utilized. This results in the production of eponemycin **1** and congeners **3**, **4**, **5** and **6**, in addition to TMC-86A **2** via the *S. hygroscopicus* pathway.

### EpnJ and EpnK play key roles in 4,5-dehydroleucine biosynthesis

Previous genetic experiments have failed to define unambiguous biosynthetic roles for *epnJ* and *epnK* (43). These genes encode an NRPS containing MbtH-like protein (MLP), adenylation (A) and peptidyl carrier protein (PCP) domains and a putative CYP, respectively. We have proposed that the homologous proteins encoded by *tmcJ* and *tmcK* could be involved in the biosynthesis of the dhL residue incorporated into TMC-86A **2** and eponemycin **1** (40).

UHPLC-ESI-Q-TOF-MS analyses showed eponemycin **1**, congeners **3**, **4**, **5** and **6**, and TMC-86A **2** are all absent from culture extracts of *S. albus* containing pCAP1000epnBGC-△*epnJ* and pCAP1000epnBGC-△*epnK* (Figure 5B, Supplementary Figure S2D). Instead, eponemycin congener **7** ([M+H]^+^*m/z* 385.2704, calculated for C_20_H_37_N_2_O_5_^+^: 385.2697), in which the hydroxymethyl group appended to the warhead and the dhL residue have been replaced by a methyl group and a l-Leu residue, respectively, is the major epoxyketone produced. This compound can also be detected in *S. albus* containing pCAP1000epnBGC. Metabolites with molecular formulae and MS/MS fragmentation patterns corresponding to dihydro-eponemycin **8** (calculated for C_20_H_37_N_2_O_6_^+^: 401.2644, measured: 401.2644), a dihydro-eponemycin congener **9** (calculated for C_21_H_35_N_2_O_6_^+^: 429.2595, measured: 429.2597), and dihydro-TMC-86A **10** (calculated for C_16_H_29_N_2_O_6_^+^: 345.2020, measured: 345.2018) were also observed (Figure 5B, Supplementary Figures S2D and S3). Introduction of pOSV556t-*epnJ* and pOSV556t-*epnK* into the corresponding mutants restored the production of eponemycin **1**, congeners **3**, **4**, **5** and **6**, and TMC-86A **2** (Supplementary Figure S2D).

These data confirm that both EpnJ and EpnK play critical roles in the biosynthesis of the dhL residue incorporated into metabolites **1**–**6**. Moreover, they are consistent with our previous proposal that the A domain of EpnJ loads l-Leu onto the downstream PCP domain and the resulting aminoacyl thioester is desaturated across C4/C5 by EpnK (40). Hydrolytic release (likely catalysed by the type II thioesterase EpnB) would afford dhL, which is incorporated into **1**–**6** by EpnG. Feeding of dhL to the *epnJ* and *epnK* mutants restored the production of **1**–**6**, consistent with this nonproteinogenic amino acid being a free intermediate in their biosynthesis (Figure 5B, Supplementary Figure S2D).

## DISCUSSION

In this work, we have developed a yeast-based platform for efficient capture and parallelized manipulation of specialized metabolite BGCs utilizing DSBs created by commercially available restriction enzymes. By combining beneficial features of the previously reported pCAP01 and pCAP03 vectors (5,12), we have created pCAP1000 containing long flanking sequences to increase the efficiency of homologous recombination, and a counter-selectable marker to reduce the number of false positives resulting from non-homologous end joining. The enhanced utility of pCAP1000 compared with pCAP01 and pCAP03 was demonstrated by comparing capture frequencies for the eponemycin and TMC-86A BGCs. No clones were obtained for either BGC using pCAP03. Using pCAP01, 23% and 0% of clones contained the eponemycin and TMC-86A BGCs, respectively. In contrast, 67% and 100% of clones contained the eponemycin and TMC-86A BGCs when pCAP1000 was used. Thus, pCAP1000 represents a useful addition to the toolkit for TAR-mediated BGC capture, particularly for cases that fail using pCAP03 due to low homologous recombination efficiency.

Yeast is an efficient tool for plasmid construction and modification due to its robust homology-directed DNA repair machinery (11,23,49,50). However, current methodology is reliant on the creation of a unique DSB at sites targeted for modification, using either a naturally occurring unique restriction site or a site-specific guide RNA in conjunction with CRISPR-Cas9 (23,50). While such methods are efficient, as illustrated by engineering of the initially captured TMC-86A BGC to incorporate the promoter region upstream of *tmcA*, creation of DSBs at specific locations can be challenging due to the random distribution of unique restriction sites and CRISPR-Cas9 digestion at off-target sites in GC-rich DNA. Our methodology circumvents these problems by exploiting the high efficiency and fidelity of homologous recombination in yeast to reassemble plasmids that are cut at multiple sites by a single restriction endonuclease. Using different combinations of repair and deletion polynucleotides enables combinatorial reassembly of plasmids containing in-frame deletions in various genes. As a proof of concept, we created six in-frame deletions in the eponemycin BGC and a single deletion in the TMC-86A BGC using various restriction enzymes producing as many as eight fragments. In all cases, the fragments were reassembled to produce BGCs containing the desired deletions. Subsequent heterologous expression, genetic and chemical complementation, and UHPLC-ESI-Q-TOF-MS analyses confirmed that all the engineered BGCs were functional and enabled biosynthetic roles to be assigned to the target genes. Thus, our methodology represents a valuable addition to the toolkit for manipulating specialized metabolite BGCs, which can be readily expanded to BGCs cloned into other vectors (such as those isolated from cosmid/fosmid libraries) by incorporating a yeast origin of replication. Moreover, using synthetic (rather than PCR-amplified) repair and deletion polynucleotides would enable this engineering platform to be automated, further facilitating parallelization. One potential limitation of our method is that reassembly efficiency could decrease with larger BGCs, because it may prove necessary to use a greater number of restriction fragments to create in-frame deletions in certain genes. This might necessitate screening of larger numbers of colonies to identify a plasmid with the intended genotype. However, in our hands, deletions can readily be constructed in captured BGCs up to 50 kb in size, and no more than 20 transformants typically needs to be screened to identify a positive clone.

**Figure 6.**
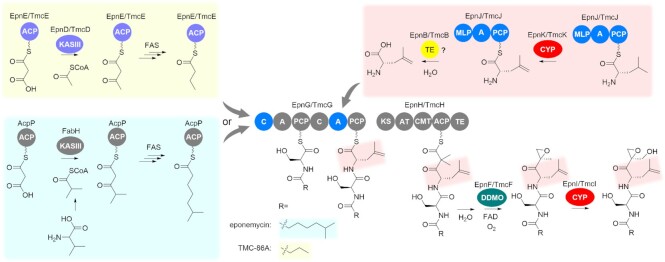
Revised biosynthetic pathway for eponemycin **1** and TMC-86A **2**. The newly identified pathways for assembly of the butanoyl thioester starter unit for TMC-86A **2** by EpnD/TmcD and EpnE/TmcE and the non-proteinogenic amino acid dhL, proposed to involve EpnK/TmcK-catalyzed desaturation of l-Leu following activation and covalent tethering by EpnJ/TmcJ are highlighted by the yellow and red boxes, respectively. The 6-methylheptanoyl thioester starter unit for eponemycin **1** biosynthesis is hypothesized to be assembled by the primary metabolic fatty acid synthase (FAS) as shown in the blue box.

The previously overlooked production of TMC-86A **2** alongside eponemycin **1** and congeners **3**–**6** when the eponemycin BGC is expressed in *S. albus*, coupled with the exclusive production of **2** when the TMC-86A BGC is introduced into this host, prompted us to investigate the biosynthetic origins of the various N-terminal fatty acyl residues in these metabolites. Our data clearly implicate the KASIII and ACP homologs encoded by *epnD/tmcD* and *epnE*/*tmcE*, respectively, in the assembly of the butanoyl thioester that initiates TMC-86A **2** biosynthesis (Figure 6). EpnD/TmcD appear to be specific for acetyl-CoA and are proposed to catalyze decarboxylative condensation of an active site-bound acetyl thioester with malonyl-EpnE/TmcE (Figure 6), mirroring the role played by KASIII homologs encoded by BGCs for other specialized metabolites (14). The resulting ACP-bound β-keto thioester is likely reduced to the corresponding butanoyl thioester by the ketoreductase, dehydratase and enoylreductase enzymes of the primary metabolic FAS. Our data also indicate that EpnD/TmcD and EpnE/TmcE play no role in assembly of the 6-methylheptanoyl thioester that initiates eponemycin **1** biosynthesis (Figure 6). This is likely assembled entirely by the primary metabolic FAS. Fatty acid biosynthesis in *Streptomyces* species is initiated primarily by FabH-catalyzed condensation of isobutyl, 2-methylbutyl and 3-methylbutyl-CoA starter units derived from l-Val, l-Ile and l-Leu, respectively, with malonyl-AcpP (51,52). Incorporation of [U-^13^C_5_]-l-valine into the N-terminal fatty acyl residue of eponemycin **1** is consistent with it being assembled by the primary metabolic FAS (43,51,52).

We previously suggested that the dhL residue of TMC-86A could be assembled by the NRPS encoded by *tmcJ* and the CYP encoded by *tmcK* (40), prior to being transferred onto the second PCP domain of TmcG (Figure 6). The data presented here are fully consistent with this proposal—TMC-86A **2** and eponemycin **1** production is abolished in *epnK* and *epnJ* mutants, but analogues containing l-Leu in place of the dhL residue are produced and feeding of dhL to the mutants restores production. Our earlier observation that both dhL and l-Leu can be adenylated by the second A domain of TmcG (40) indicates that dhL is hydrolytically released after EpnK/TmcK-catalyzed desaturation of the l-Leu residue bound to the PCP domain of EpnG/TmcG. The type II thioesterase (TE) encoded by *epnB*/*tmcB* is likely to catalyze dhL release (Figure 6). However, this hypothesis is difficult to verify genetically, because deletion of genes encoding type II TEs is known to dramatically impact metabolite production levels in several NRPS and PKS systems (53,54). Intriguingly, the synthetic epoxyketone KZR-616, which selectively targets the immunoproteasome and is currently in phase II trials for lupus nephritis (55), contains a l-(1-cyclopentenyl)alanine (CPA) residue in the position corresponding to the dhL residue in eponemycin **1** and TMC-86A **2**. It will therefore be interesting to investigate whether the biosynthetic machinery encoded by the eponemycin and TMC-86A BGCs is able to incorporate CPA instead of dhL into novel epoxyketone derivatives.

In conclusion, we have developed a robust and versatile platform for efficient cloning and parallelized manipulation of BGCs that is expected to find wide-spread application in elucidating mechanisms of specialized metabolite biosynthesis. Application of this platform to the eponemycin and TMC-86A BGCs has illuminated key steps in the assembly of these metabolites and lays the foundation for future biosynthetic engineering efforts directed toward the production of clinically useful epoxyketones.

## DATA AVAILABILITY

All data needed to interpret, verify, and extend the research are provided in the main manuscript and supplementary data. The raw LC–MS data, which were processed via standard extracted ion chromatogram procedures, are available upon written request to the corresponding author.

## Supplementary Material

gkad009_Supplemental_File

## Notes

Present address: Daniel Zabala, Faes Farma, Bilbao, Spain.

Present address: Emmanuel L.C. de los Santos, UCB Celltech, Slough, Berkshire SL1 3WE, UK.
