# Supplementary material for: Parallelized gene cluster editing illuminates mechanisms of epoxyketone proteasome inhibitor biosynthesis
Source: Nucleic Acids Res. 2023 Jan 31;51(3):1488–99. doi: 10.1093/nar/gkad009 (PMC9943649; doi:10.1093/nar/gkad009)
Supplement: gkad009_Supplemental_File [file gkad009_supplemental_file.pdf]

Supplementary Data for

Parallelized gene cluster editing illuminates mechanisms of epoxyketone proteasome inhibitor biosynthesis.

Chuan Huang[1,2,4,5*], Daniel Zabala[1], Emmanuel L. C. de los Santos[1,2,3], Lijiang Song[1], Christophe Corre[1,2,3], Lona M. Alkhalaf[1], and Gregory L. Challis[1,2,4,5*]

[1]Department of Chemistry, University of Warwick, Coventry CV4 7AL, UK
[2]Warwick Integrative Synthetic Biology Centre, University of Warwick, Coventry CV4 7AL, UK
[3]School of Life Sciences, University of Warwick, Coventry CV4 7AL, UK
[4]Biomedicine Discovery Institute, Department of Biochemistry and Molecular Biology, Monash University, Clayton, Victoria 3800, Australia
[5]ARC Centre of Excellence for Innovations in Peptide and Protein Science, Monash University, Clayton, Victoria 3800, Australia

* Chuan Huang. Tel: +61 03 9905 1750; Email: chuan.huang@monash.edu

* Gregory L. Challis. Tel: +44 02476 522355; Email: g.l.challis@warwick.ac.uk

This PDF file contains:

Figures S1 to S4
Tables S1 to S4
References

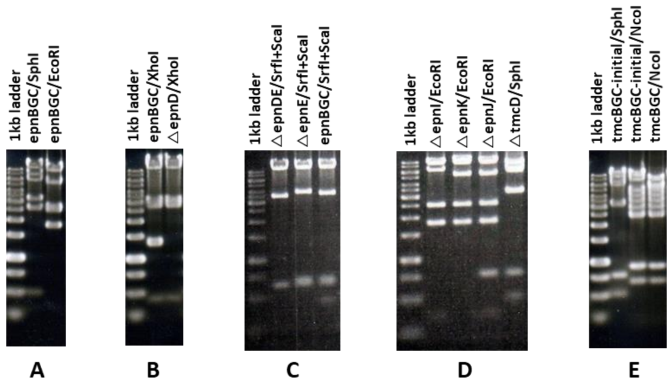

	Plasmid/Restriction enzyme	Expected size of restriction fragments
A.	pCAP1000epnBGC/EcoRI	101, 10032, 1808, 5633, 2646, 309, 12228
	pCAP1000epnBGC/SphI	12037, 2614, 271, 488, 3295, 6236, 7906
B.	pCAP1000epnBGC/XhoI	1374, 68, 2829, 399, 3129, 12695, 12870
	pCAP1000epnBGC-△epnD/XhoI	68, 2829, 399, 3129, 12695, 13233
C.	pCAP1000epnBGC-△epnDE/SrfI+ScaI	3618, 13282, 635, 14592
	pCAP1000epnBGC-△epnE/SrfI+ScaI	686, 3988, 13282, 635, 14593
	pCAP1000epnBGC/SrfI+ScaI	686, 426, 3748, 13279, 635, 14593
D	pCAP1000epnBGC-△epnI/EcoRI	2498, 101, 10032, 1808, 7091, 309, 10337
	pCAP1000epnBGC-△epnK/EcoRI	2498, 101, 10032, 1808, 5633, 12212
	pCAP1000epnBGC-△epnJ/EcoRI	2499, 101, 10033, 1811, 5633, 762, 309, 10338
	pCAP1000tmcBGC-△tmcD/SphI	7169, 763, 6299, 3235, 488, 9222, 3261, 7135
E	pCAP1000tmcBGC-initial/SphI	7169, 763, 6299, 3235, 488, 10206, 9401
	pCAP1000tmcBGC-initial/NcoI	3369, 2840, 8116, 900, 4816, 2440, 666, 4046, 147, 159, 10062
	pCAP1000tmcBGC/NcoI	3369, 2840, 8116, 900, 4816, 2440, 666, 4046, 147, 159, 5395, 5662

Fig. S1. Restriction enzyme digest data for pCAP1000epnBGC, pCAP1000tmcBGC-initial and derivatives. (A) pCAP1000epnBGC SphI and EcoRI digest. (B) Comparison of pCAP1000epnBGC-△epnD and pCAP1000epnBGC XhoI digests. (C) Comparison of pCAP1000epnBGC, pCAP1000epnBGC-△epnE and pCAP1000epnBGC-△epnDE SrfI and ScaI double digests. (D) EcoRI digests of pCAP1000epnBGC-△epnI, pCAP1000epnBGC-△epnK and pCAP1000epnBGC-△epnJ and SphI digest of pCAP1000tmcBGC-△tmcD. (E) Comparison of NcoI digests for pCAP1000tmcBGC-initial and pCAP1000tmcBGC, and SphI digest for pCAP1000tmcBGC-initial.

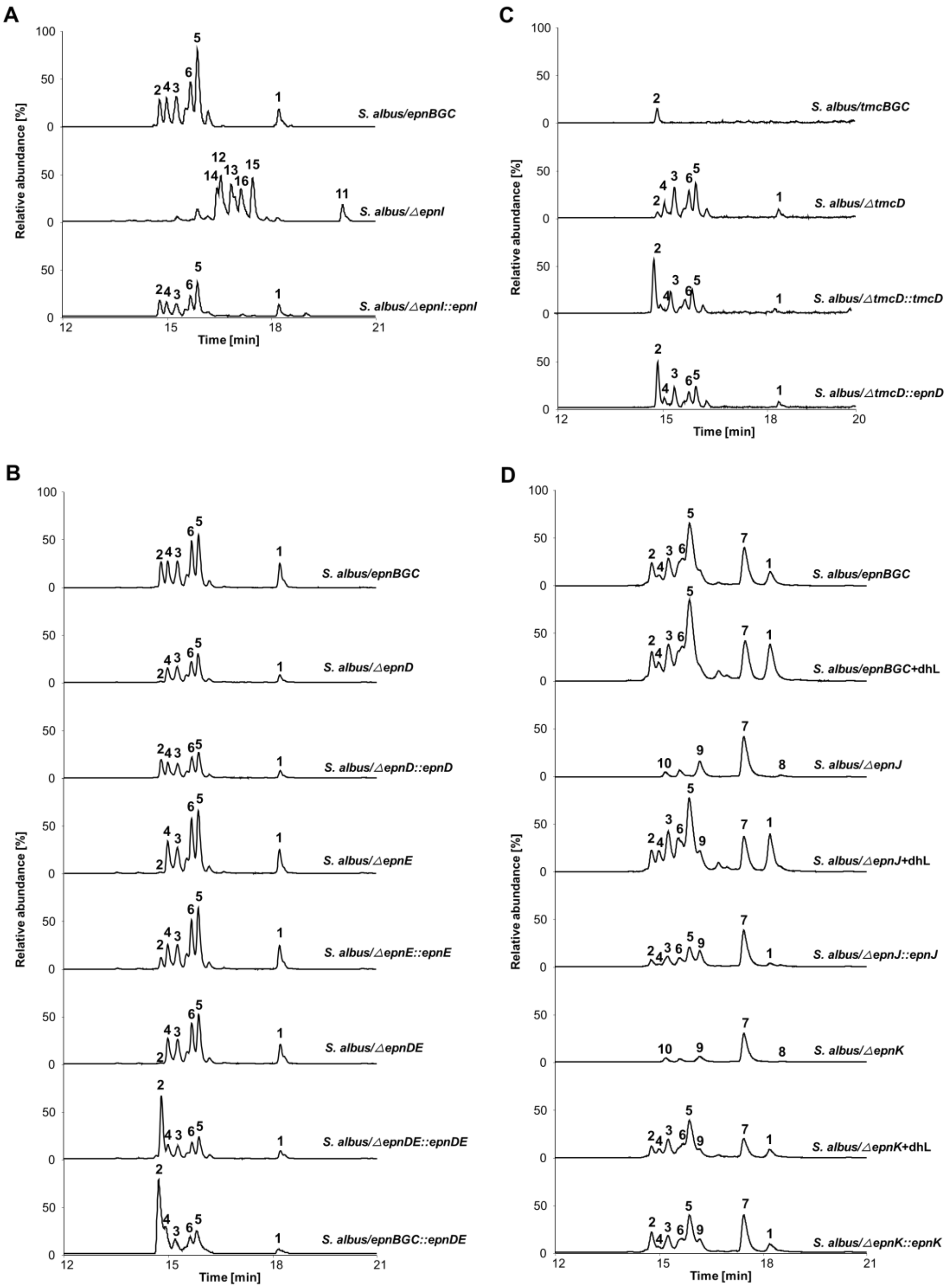

Fig. S2. Extracted ion chromatograms (EICs) from UHPLC-ESI-Q-TOF-MS analyses of culture extracts from *S. albus* containing pCAP1000epnBGC, pCAP1000tmcBGC and various engineered derivatives. (A) EICs at *m/z* = 421.2309 ± 0.002, 365.1683 ± 0.002, 423.2466 ± 0.002, 449.2258 ± 0.002, 451.2415 ± 0.002, 435.2102 ± 0.002, 437.2258 ± 0.002, 433.2309 ± 0.002, 435.2466 ± 0.002, 419.2153 ± 0.002, 349.1734 ± 0.002, and 405.2360 ± 0.002, corresponding to $[M+Na]^+$ for eponemycin **1** (and isomeric deshydroxy-eponemycin congener **14**), TMC-86A **2**, dihydro-eponomycin **8**, eponemycin congener **5**, eponemycin congener **6**, eponemycin congener **3**, eponemycin congener **4**, deshydroxy-eponemycin congener **15,**

deshydroxy eponemycin congener **16**, deshydroxy-eponemycin congener **13**, deshydroxy-TMC-86A **12** and deshydroxy-eponemycin **11**, for *S. albus* containing pCAP1000epnBGC; the Δ*epnI* derivative; and Δ*epnI* derivative plus *epnI* under the control of the constitutive *ermE** promoter. (B) EICs at *m/z* = 421.2309 ± 0.002, 365.1683 ± 0.002, 435.2102 ± 0.002, 437.2258 ± 0.002, 449.2258 ± 0.002, and 451.2415 ± 0.002, corresponding to [M+Na]+ for eponemycin **1**, TMC-86A **2**, eponemycin congener **3**, eponemycin congener **4**, eponemycin congener **5** and eponemycin congener **6** for *S. albus* containing pCAP1000epnBGC; Δ*epnD*, Δ*epnE* and Δ*epnDE* derivatives; Δ*epnD*, Δ*epnE* and Δ*epnDE* derivatives plus *epnD*, *epnE* and *epnDE* (respectively) under the control of the constitutive *ermE** promoter; and pCAP1000epnBGC plus *epnDE* under the control of the *ermE** promoter. (C) EICs at *m/z* = 421.2309 ± 0.002, 365.1683 ± 0.002, 435.2102 ± 0.002, 437.2258 ± 0.002, 449.2258 ± 0.002, and 451.2415 ± 0.002, corresponding to [M+Na]+ for eponemycin **1**, TMC-86A **2**, eponemycin congener **3**, eponemycin congener **4**, eponemycin congener **5** and eponemycin congener **6** for *S. albus* containing pCAP1000tmcBGC, a Δ*tmcD* derivative, and the Δ*tmcD* derivative containing *tmcD* or *epnD* under the control of the constitutive *ermE** promoter. (D) EICs at *m/z* = 421.2309 ± 0.002, 365.1683 ± 0.002, 407.2516± 0.002, 435.2102 ± 0.002, 437.2258 ± 0.002, 449.2258 ± 0.002, 451.2415 ± 0.002, 367.1845 ± 0.002, 423.2466 ± 0.002, corresponding to $[M+Na]^+$ for eponemycin **1**, TMC-86A **2**, deshydroxy-dehydro-eponemycin congener **7**, eponemycin congener **3**, eponemycin congener **4**, eponemycin congener **5**, eponemycin congener **6** (and the isomeric dihydro-eponomycin congener **9**), dihydro-TMC-86A **10** and dihydro-eponemycin **8** for *S. albus* containing pCAP1000epnBGC with and without addition of exogenous dhL, Δ*epnK* and Δ*epnJ* with and without addition of exogenous dhL and the Δ*epnK* and Δ*epnJ* derivatives containing *epnK* and *epnJ,* respectively*,* under the control of the constitutive *ermE** promoter.

A

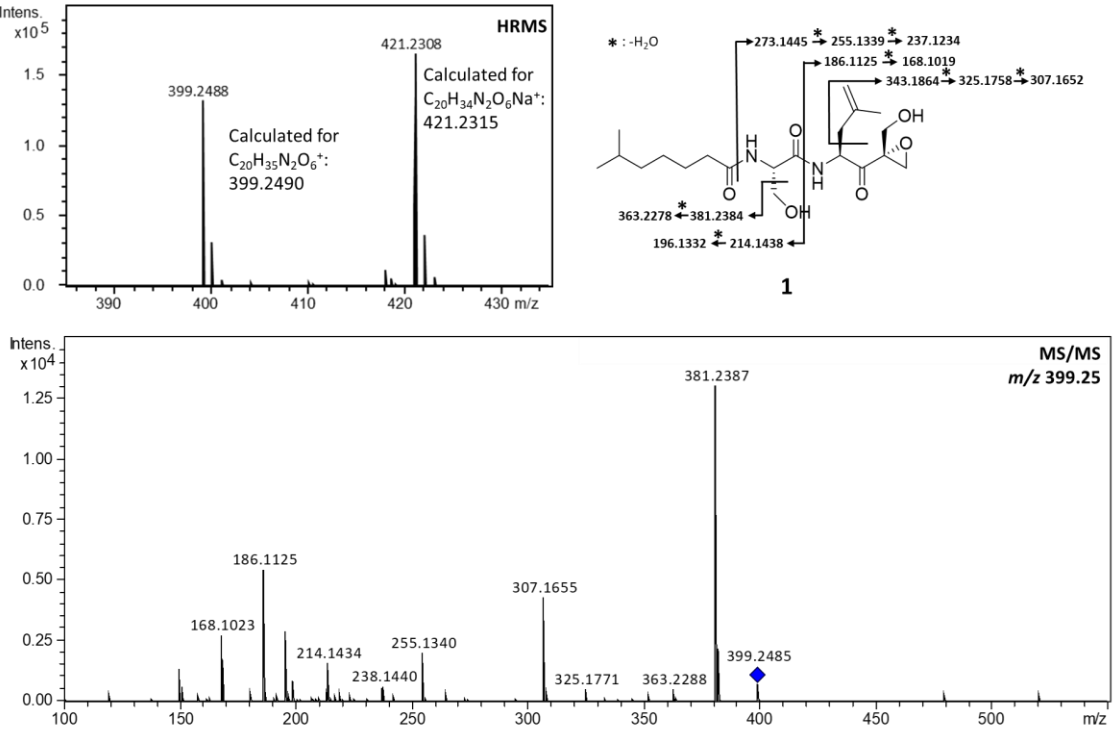
HRMS
Intens. x10^5
399.2488
421.2308
Calculated for $C_{20}H_{35}N_2O_6^+$: 399.2490
Calculated for $C_{20}H_{34}N_2O_6Na^+$: 421.2315
m/z
* : $-H_2O$
273.1445 → 255.1339 → 237.1234
186.1125 → 168.1019
343.1864 → 325.1758 → 307.1652
363.2278 ← 381.2384
196.1332 ← 214.1438
1
MS/MS
m/z 399.25
381.2387
186.1125
307.1655
168.1023
214.1434
255.1340
238.1440
325.1771
363.2288
399.2485

B

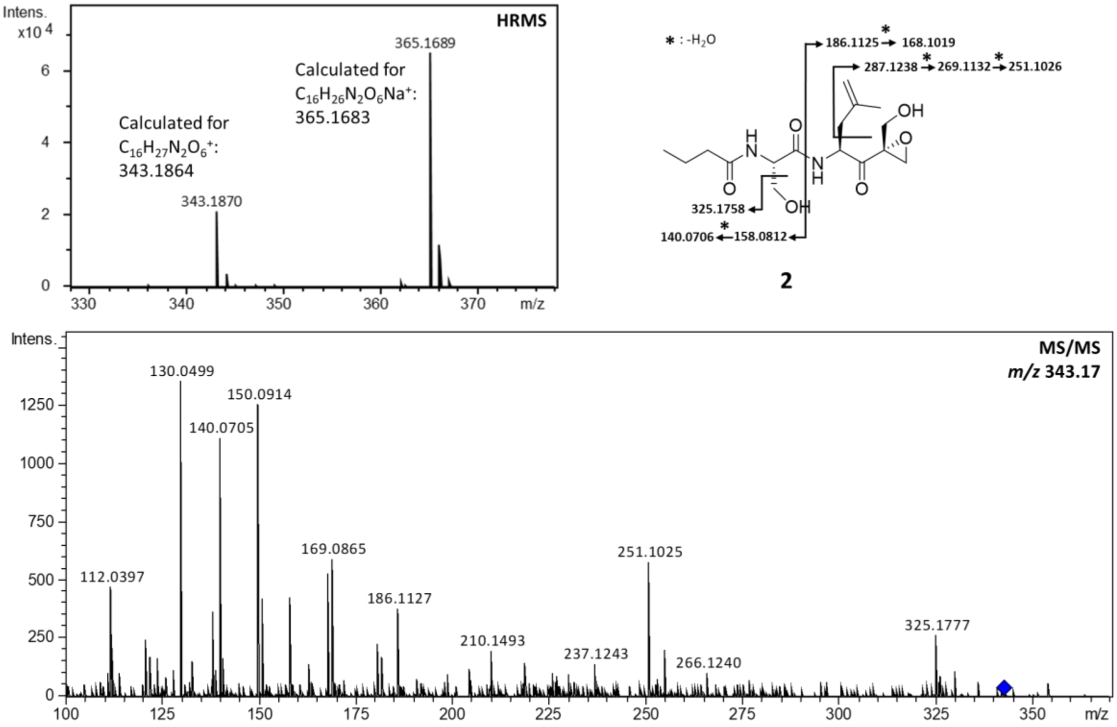
HRMS
Intens. x10^4
365.1689
Calculated for $C_{16}H_{26}N_2O_6Na^+$: 365.1683
Calculated for $C_{16}H_{27}N_2O_6^+$: 343.1864
343.1870
m/z
* : $-H_2O$
186.1125 → 168.1019
287.1238 → 269.1132 → 251.1026
325.1758
140.0706 ← 158.0812
2
MS/MS
m/z 343.17
130.0499
150.0914
140.0705
169.0865
112.0397
186.1127
251.1025
210.1493
237.1243
266.1240
325.1777

C

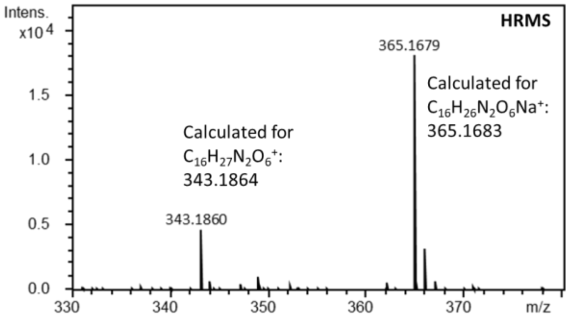
HRMS
Intens.
x10^4
365.1679
Calculated for $C_{16}H_{26}N_2O_6Na^+$: 365.1683
Calculated for $C_{16}H_{27}N_2O_6^+$: 343.1864
343.1860
m/z

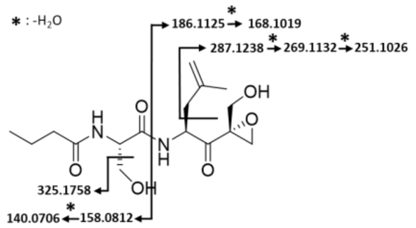
* : -H_2O
186.1125 → 168.1019
287.1238 → 269.1132 → 251.1026
OH
325.1758
140.0706 ← 158.0812

2

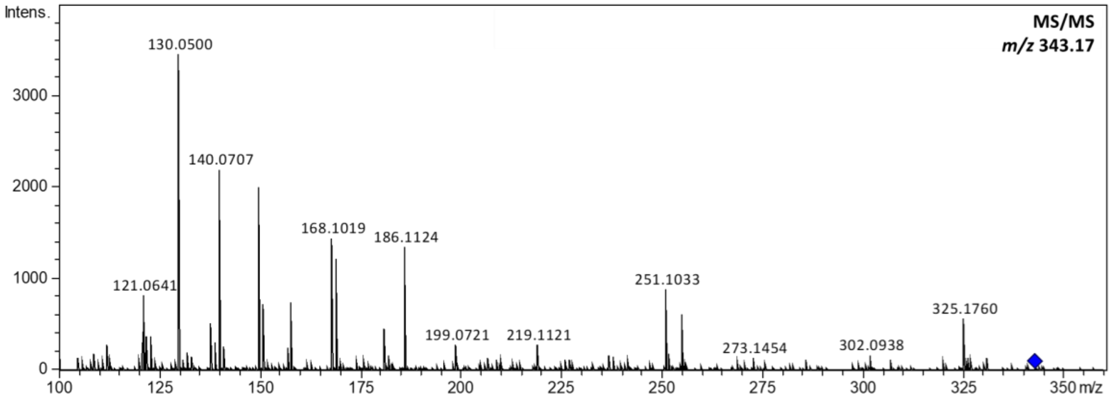
MS/MS
m/z 343.17
Intens.
130.0500
140.0707
168.1019
186.1124
121.0641
251.1033
325.1760
199.0721
219.1121
273.1454
302.0938
m/z

D

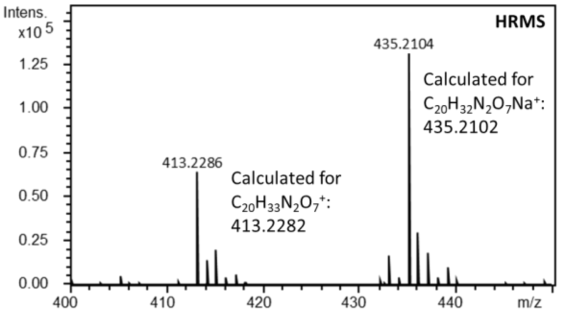
HRMS
Intens.
x10^5
435.2104
Calculated for $C_{20}H_{32}N_2O_7Na^+$: 435.2102
413.2286
Calculated for $C_{20}H_{33}N_2O_7^+$: 413.2282
m/z

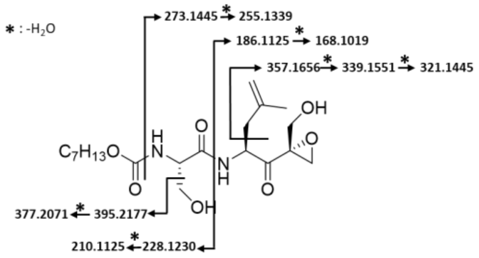
* : -H_2O
273.1445 → 255.1339
186.1125 → 168.1019
357.1656 → 339.1551 → 321.1445
$C_7H_{13}O$
OH
377.2071 ← 395.2177
210.1125 ← 228.1230

3

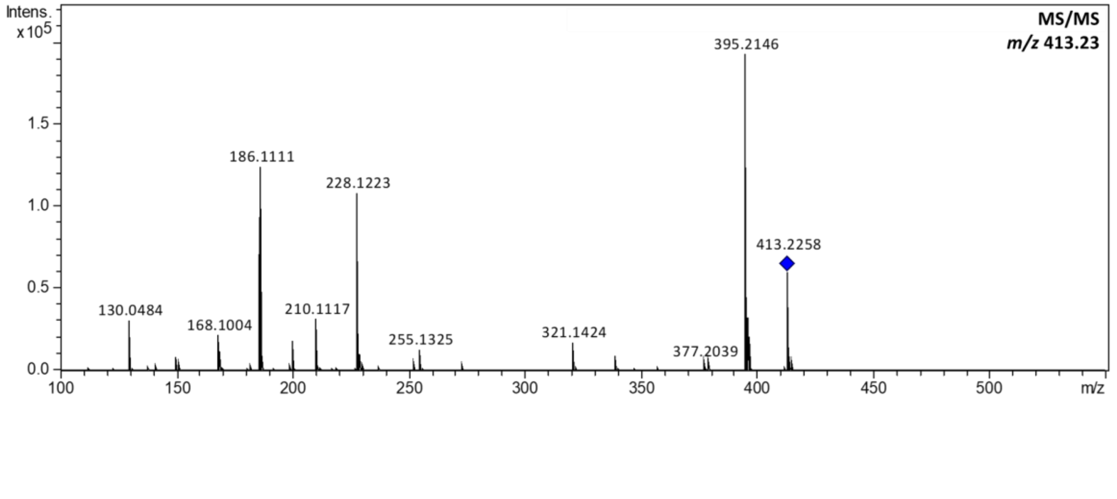
MS/MS
m/z 413.23
Intens.
x10^5
395.2146
186.1111
228.1223
413.2258
130.0484
168.1004
210.1117
255.1325
321.1424
377.2039
m/z

E

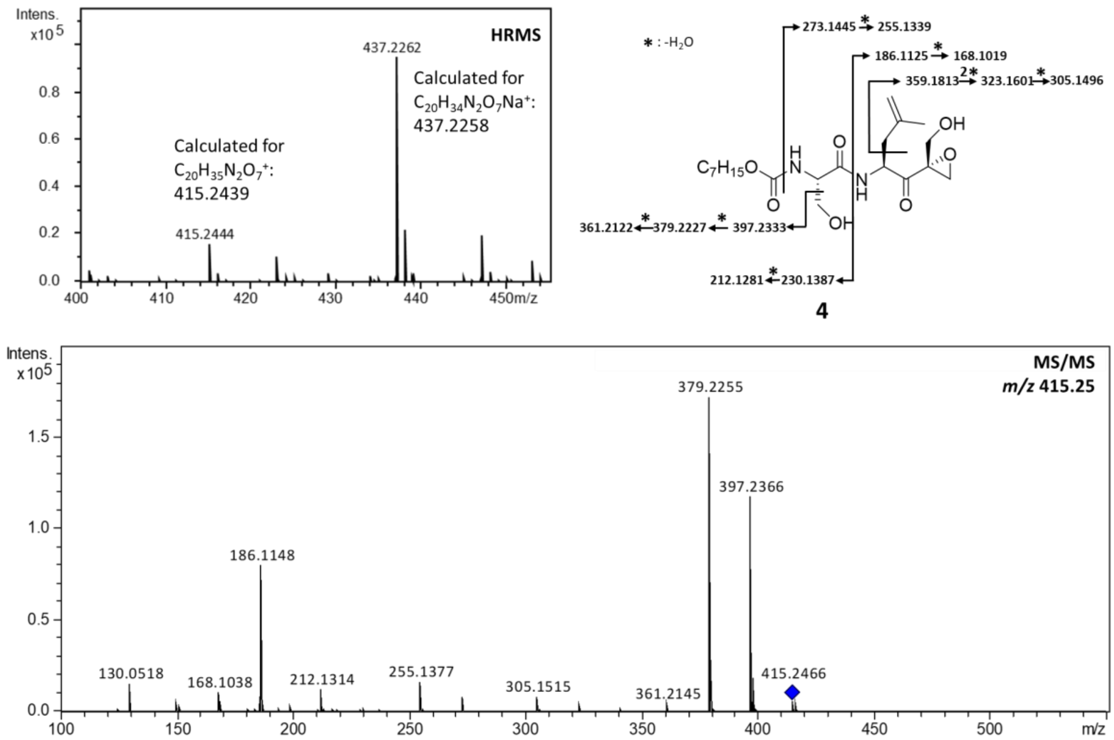

HRMS
Intens. x10^5
437.2262
Calculated for $C_{20}H_{34}N_2O_7Na^+$: 437.2258
Calculated for $C_{20}H_{35}N_2O_7^+$: 415.2439
415.2444
m/z
* : -H_2O
273.1445 * 255.1339
186.1125 * 168.1019
359.1813 2* 323.1601 * 305.1496
361.2122 * 379.2227 * 397.2333
212.1281 * 230.1387
$C_7H_{15}O$
OH
4
MS/MS
m/z 415.25
379.2255
397.2366
186.1148
130.0518
168.1038
212.1314
255.1377
305.1515
361.2145
415.2466

F

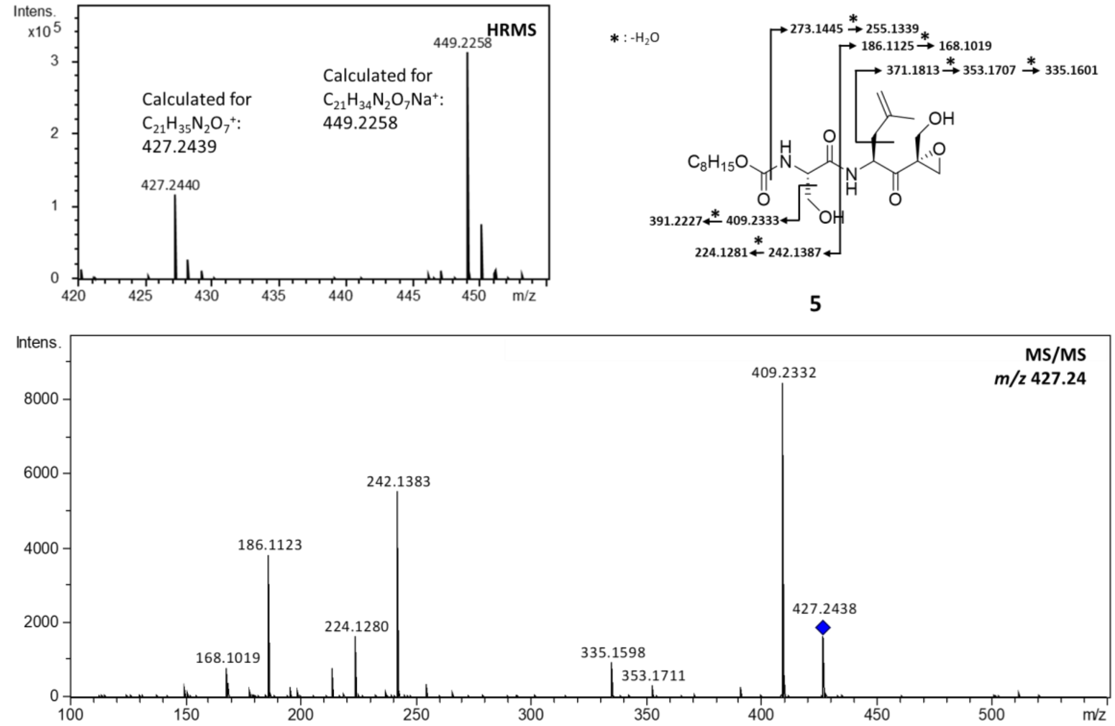

HRMS
Intens. x10^5
449.2258
Calculated for $C_{21}H_{34}N_2O_7Na^+$: 449.2258
Calculated for $C_{21}H_{35}N_2O_7^+$: 427.2439
427.2440
m/z
* : -H_2O
273.1445 * 255.1339
186.1125 * 168.1019
371.1813 * 353.1707 * 335.1601
391.2227 * 409.2333
224.1281 * 242.1387
$C_8H_{15}O$
OH
5
MS/MS
m/z 427.24
409.2332
242.1383
186.1123
224.1280
168.1019
335.1598
353.1711
427.2438

G

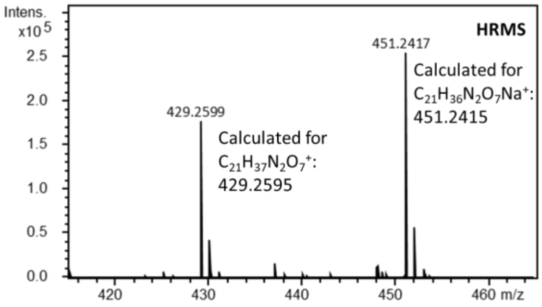
Intens.
x10^5
HRMS
451.2417
Calculated for
$C_{21}H_{36}N_2O_7Na^+$:
451.2415
429.2599
Calculated for
$C_{21}H_{37}N_2O_7^+$:
429.2595
m/z

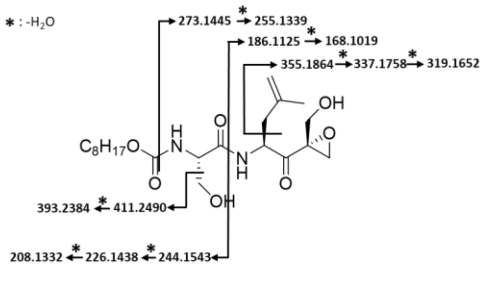
*: $-H_2O$
273.1445 * 255.1339
186.1125 * 168.1019
355.1864 * 337.1758 * 319.1652
$C_8H_{17}O$
OH
393.2384 * 411.2490
208.1332 * 226.1438 * 244.1543

6

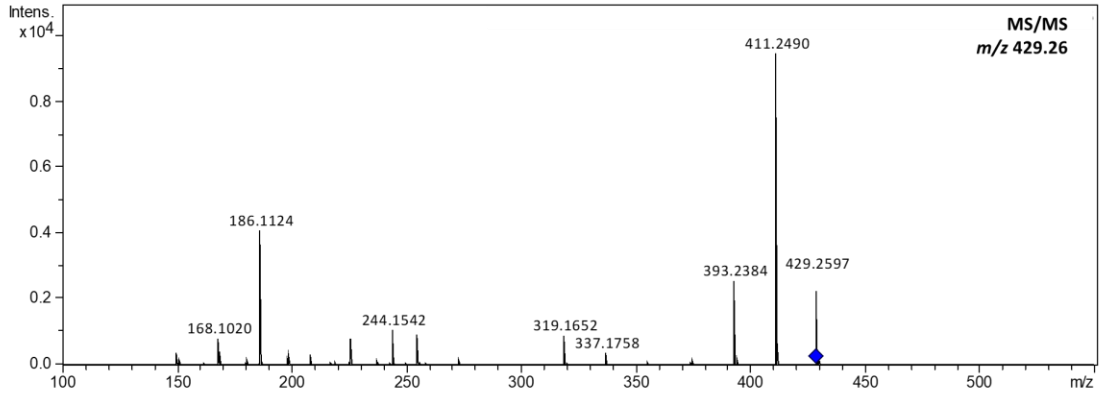
Intens.
x10^4
MS/MS
m/z 429.26
411.2490
186.1124
393.2384
429.2597
168.1020
244.1542
319.1652
337.1758
m/z

H

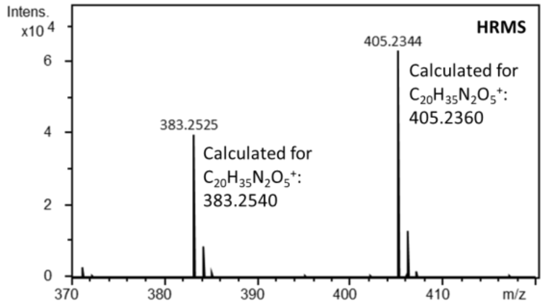
Intens.
x10^4
HRMS
405.2344
Calculated for
$C_{20}H_{35}N_2O_5^+$:
405.2360
383.2525
Calculated for
$C_{20}H_{35}N_2O_5^+$:
383.2540
m/z

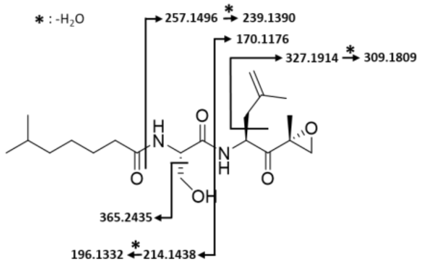
*: $-H_2O$
257.1496 * 239.1390
170.1176
327.1914 * 309.1809
OH
365.2435
196.1332 * 214.1438

11

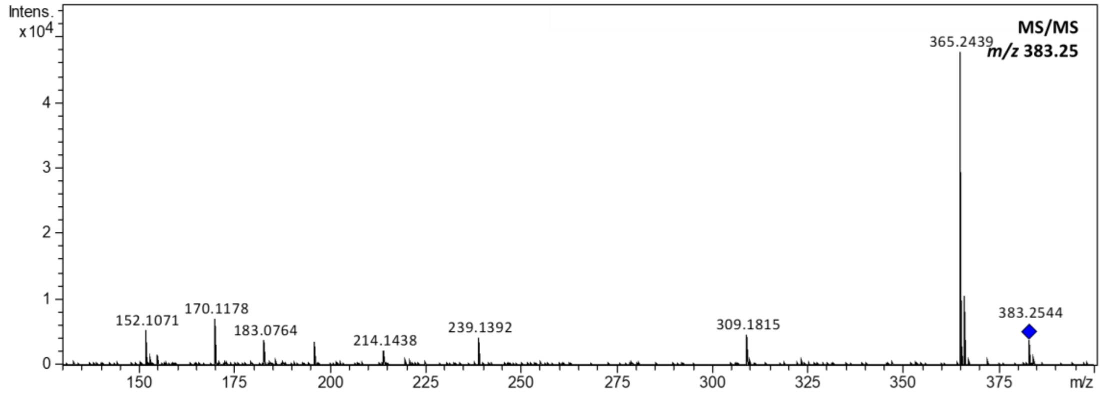
Intens.
x10^4
MS/MS
m/z 383.25
365.2439
383.2544
152.1071
170.1178
183.0764
214.1438
239.1392
309.1815
m/z

I

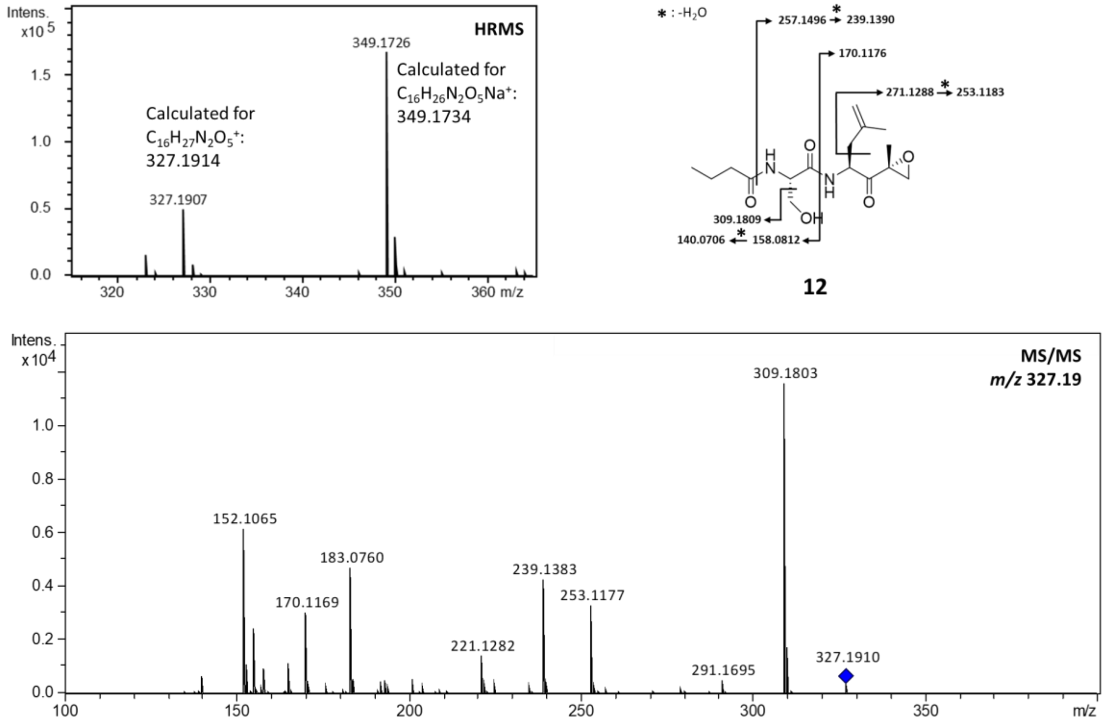
Intens.
x10 5
HRMS
349.1726
Calculated for
$C_{16}H_{26}N_2O_5Na^+$:
349.1734
Calculated for
$C_{16}H_{27}N_2O_5^+$:
327.1914
327.1907
m/z
* : -H_2O
257.1496 * 239.1390
170.1176
271.1288 * 253.1183
309.1809
140.0706 * 158.0812
12
Intens.
x10 4
MS/MS
m/z 327.19
309.1803
152.1065
183.0760
170.1169
239.1383
253.1177
221.1282
291.1695
327.1910
m/z

J

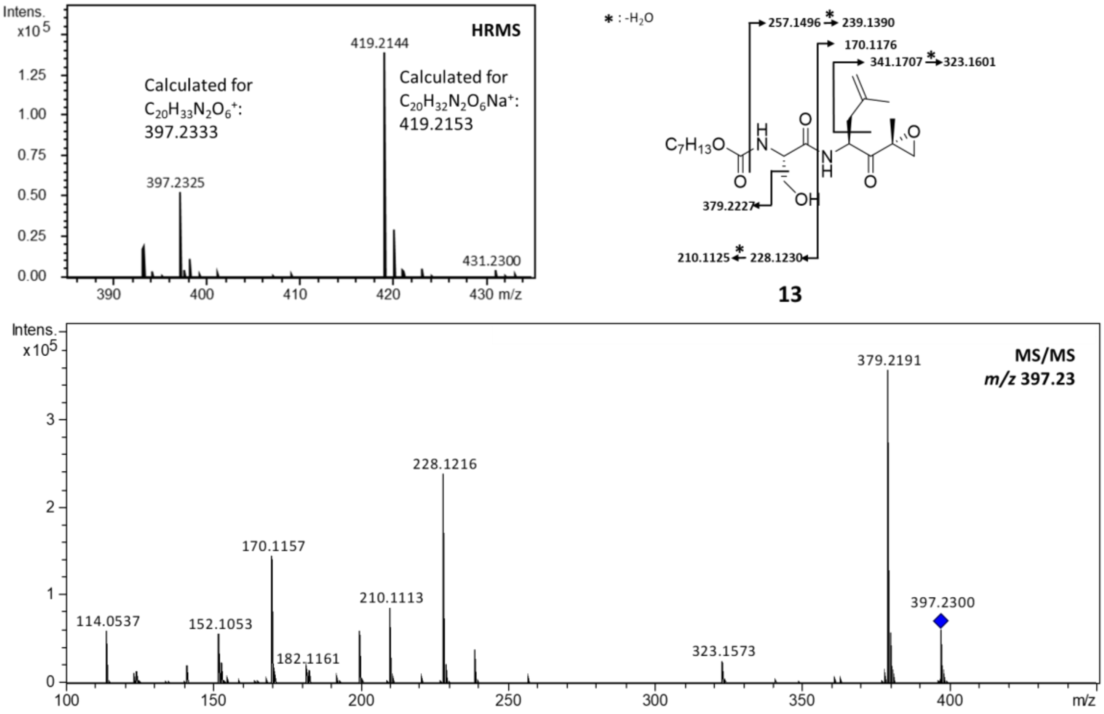
Intens.
x10 5
HRMS
419.2144
Calculated for
$C_{20}H_{33}N_2O_6^+$:
397.2333
Calculated for
$C_{20}H_{32}N_2O_6Na^+$:
419.2153
397.2325
431.2300
m/z
* : -H_2O
257.1496 * 239.1390
170.1176
341.1707 * 323.1601
$C_7H_{13}O$
379.2227
210.1125 * 228.1230
13
Intens.
x10 5
MS/MS
m/z 397.23
379.2191
228.1216
170.1157
210.1113
114.0537
152.1053
182.1161
323.1573
397.2300
m/z

K

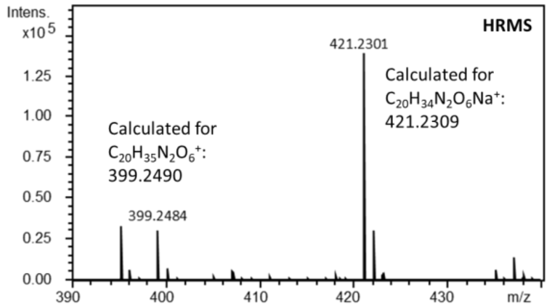
Intens.
x10^5
HRMS
421.2301
Calculated for
$C_{20}H_{34}N_2O_6Na^+$:
421.2309
Calculated for
$C_{20}H_{35}N_2O_6^+$:
399.2490
399.2484
m/z

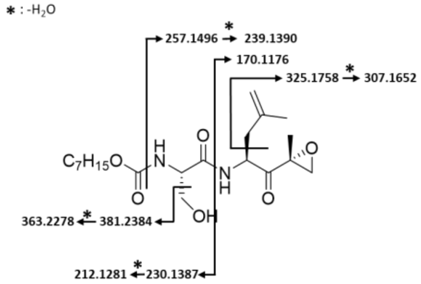
* : -H_2O
257.1496 * 239.1390
170.1176
325.1758 * 307.1652
$C_7H_{15}O$
363.2278 * 381.2384
212.1281 * 230.1387

14

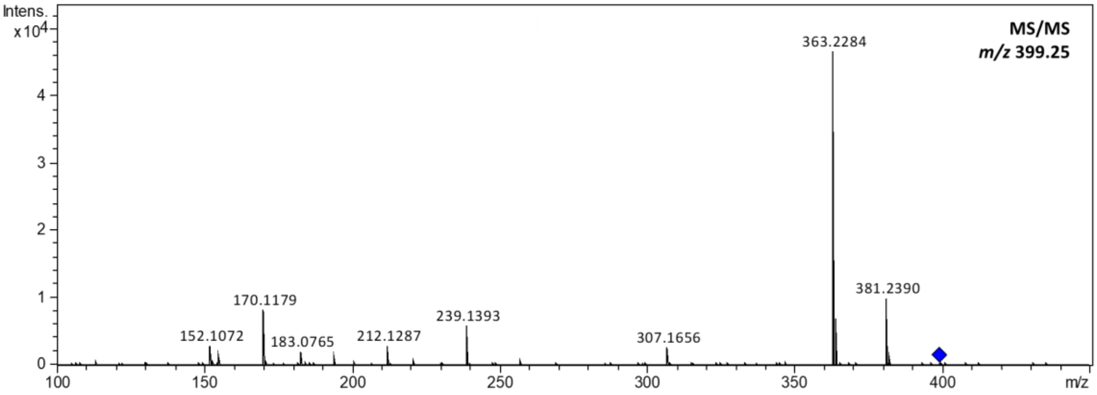
Intens.
x10^4
MS/MS
m/z 399.25
363.2284
381.2390
170.1179
152.1072
183.0765
212.1287
239.1393
307.1656
m/z

L

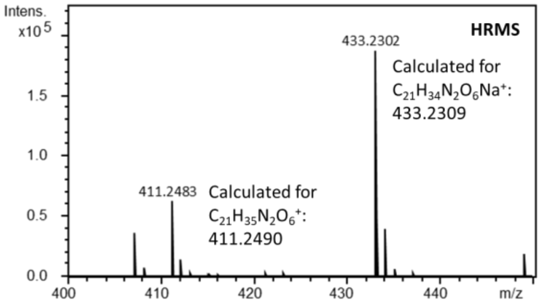
Intens.
x10^5
HRMS
433.2302
Calculated for
$C_{21}H_{34}N_2O_6Na^+$:
433.2309
411.2483
Calculated for
$C_{21}H_{35}N_2O_6^+$:
411.2490
m/z

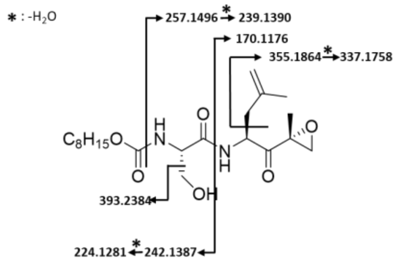
* : -H_2O
257.1496 * 239.1390
170.1176
355.1864 * 337.1758
$C_8H_{15}O$
393.2384
224.1281 * 242.1387

15

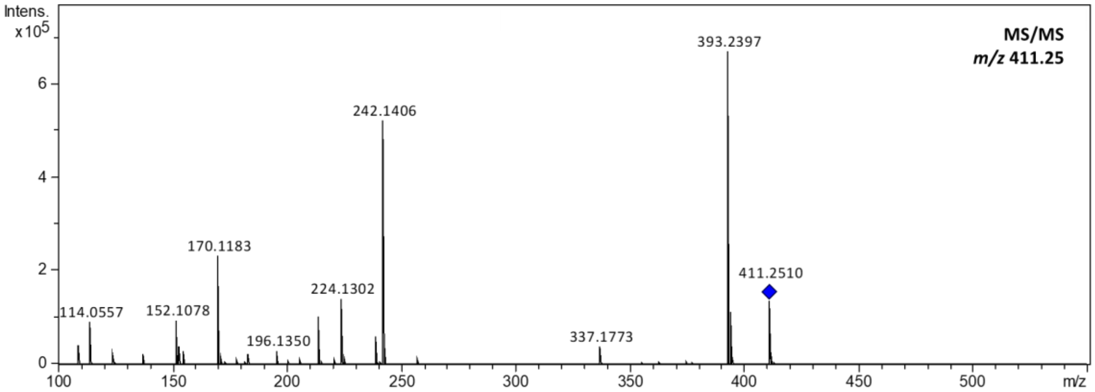
Intens.
x10^5
MS/MS
m/z 411.25
393.2397
242.1406
170.1183
411.2510
224.1302
114.0557
152.1078
196.1350
337.1773
m/z

M

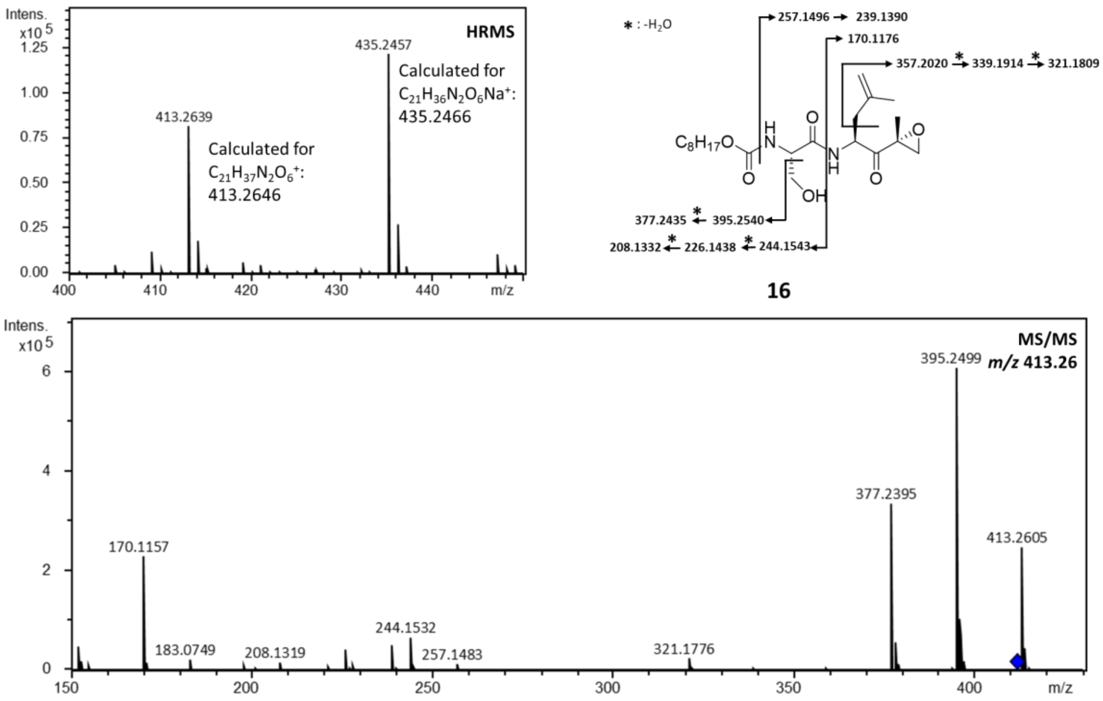
HRMS
Intens. x10^5
435.2457
Calculated for $C_{21}H_{36}N_2O_6Na^+$: 435.2466
413.2639
Calculated for $C_{21}H_{37}N_2O_6^+$: 413.2646
m/z
* : $-H_2O$
257.1496 → 239.1390
170.1176
357.2020 * 339.1914 * 321.1809
$C_8H_{17}O$
OH
377.2435 * 395.2540
208.1332 * 226.1438 * 244.1543
16
MS/MS
m/z 413.26
395.2499
377.2395
413.2605
170.1157
183.0749
208.1319
244.1532
257.1483
321.1776

N

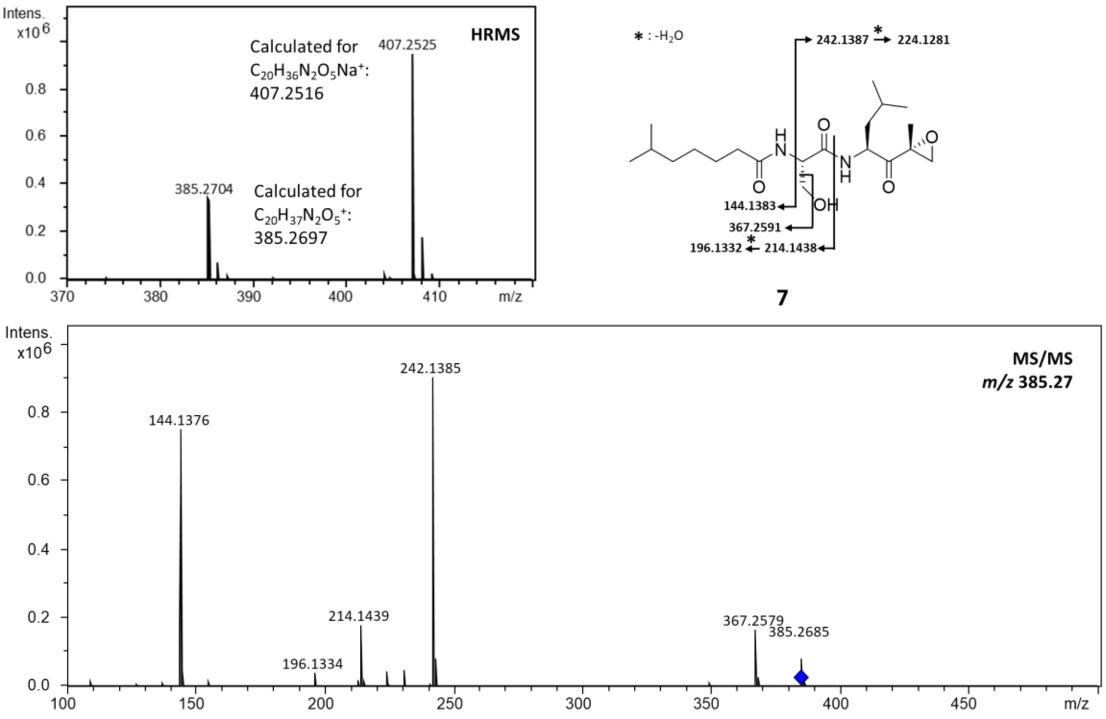
HRMS
Intens. x10^6
Calculated for $C_{20}H_{36}N_2O_5Na^+$: 407.2516
407.2525
385.2704
Calculated for $C_{20}H_{37}N_2O_5^+$: 385.2697
m/z
* : $-H_2O$
242.1387 * 224.1281
144.1383
367.2591
196.1332 * 214.1438
OH
7
MS/MS
m/z 385.27
242.1385
144.1376
214.1439
196.1334
367.2579
385.2685

O

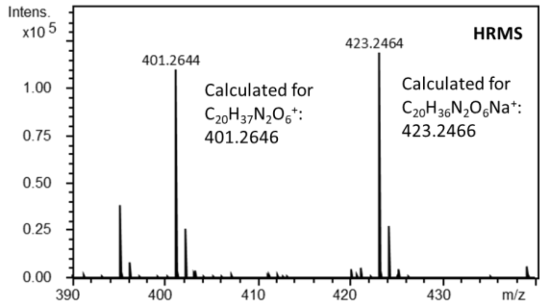
Intens.
x10 5
HRMS
401.2644
423.2464
Calculated for $C_{20}H_{37}N_2O_6^+$: 401.2646
Calculated for $C_{20}H_{36}N_2O_6Na^+$: 423.2466
m/z

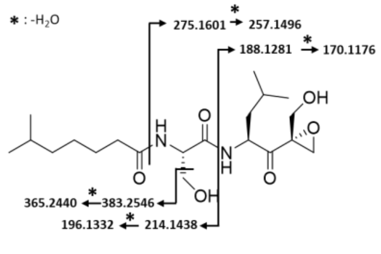
* : -H_2O
275.1601 → 257.1496
188.1281 → 170.1176
365.2440 ← 383.2546
196.1332 ← 214.1438
OH
O
H
N

8

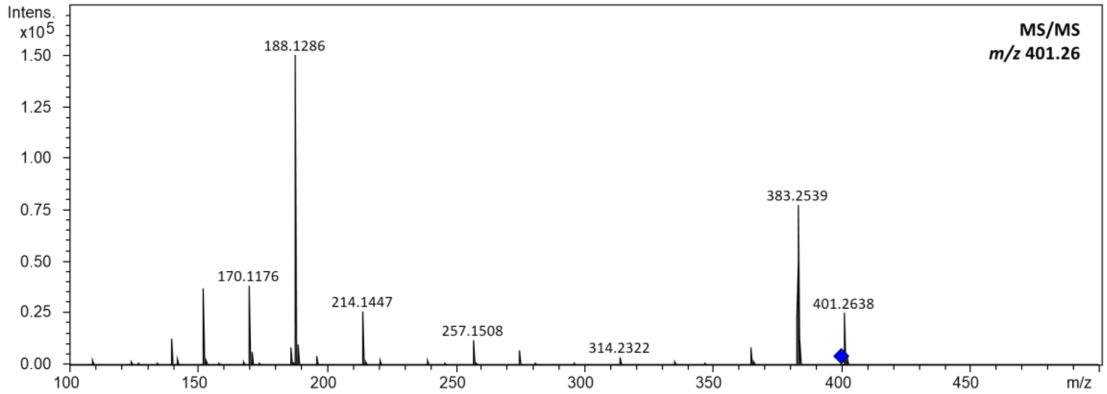
Intens.
x10 5
MS/MS
m/z 401.26
188.1286
383.2539
170.1176
214.1447
257.1508
314.2322
401.2638
m/z

P

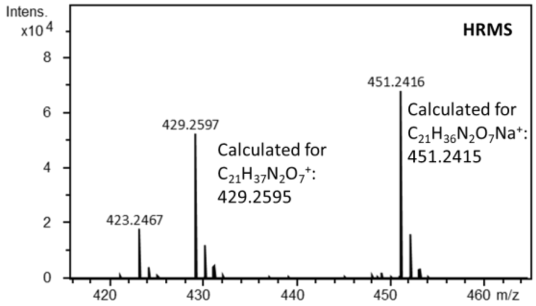
Intens.
x10 4
HRMS
451.2416
429.2597
423.2467
Calculated for $C_{21}H_{37}N_2O_7^+$: 429.2595
Calculated for $C_{21}H_{36}N_2O_7Na^+$: 451.2415
m/z

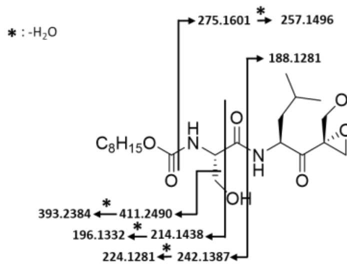
* : -H_2O
275.1601 → 257.1496
188.1281
$C_8H_{15}O$
393.2384 ← 411.2490
196.1332 ← 214.1438
224.1281 ← 242.1387
OH

9

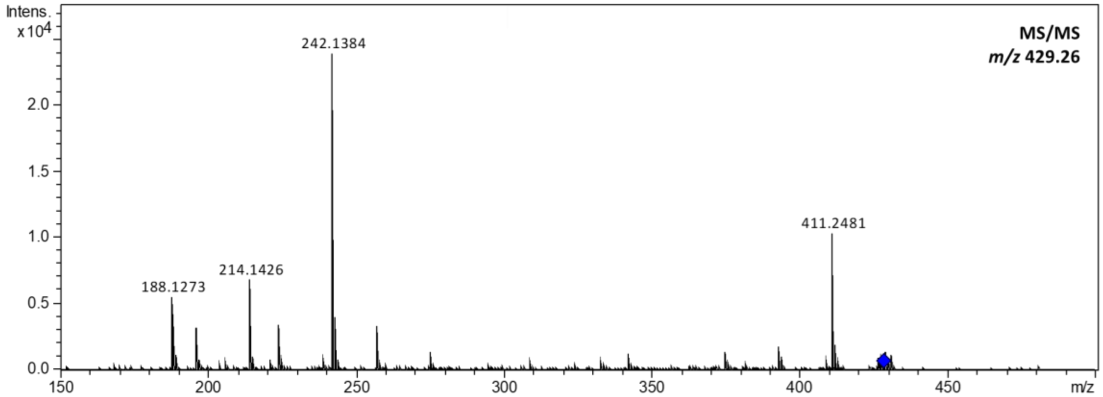
Intens.
x10 4
MS/MS
m/z 429.26
242.1384
411.2481
188.1273
214.1426
m/z

Q

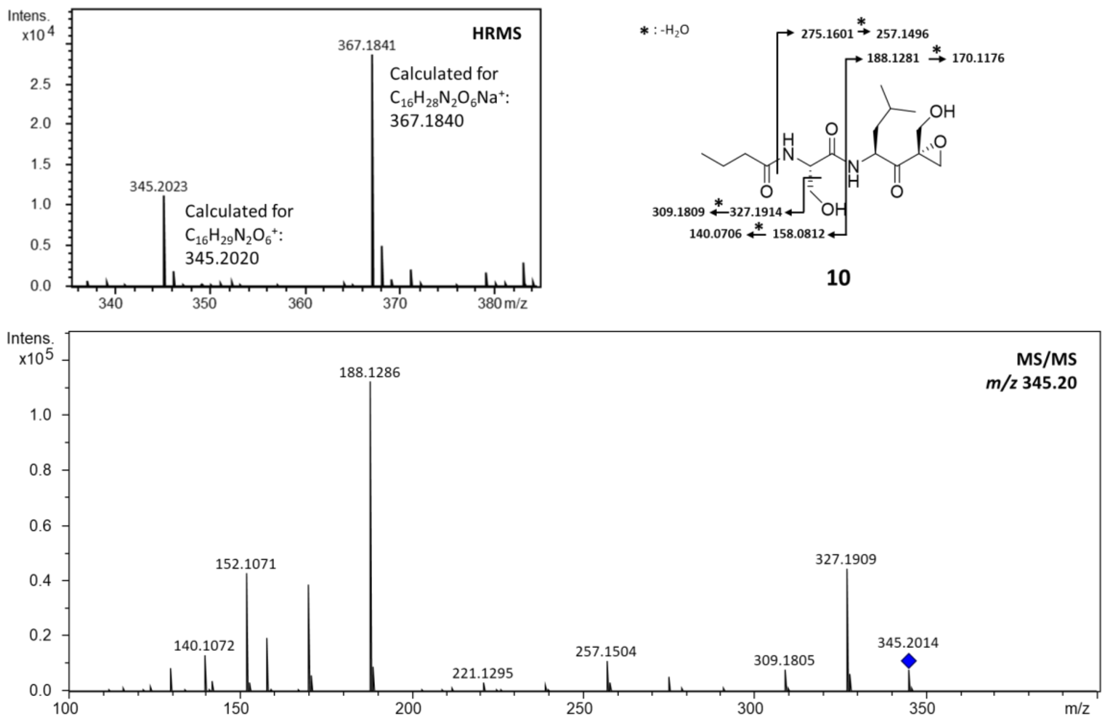

Fig. S3. High resolution and tandem mass spectra from UHPLC-ESI-Q-TOF-MS/MS analyses of culture extracts from *S. albus* containing pCAP1000epnBGC, pCAP1000tmcBGC and various engineered derivatives. (A) Eponemycin **1** in extracts of *S. albus* containing pCAP1000epnBGC. (B) TMC-86A **2** in extracts of *S. albus* containing pCAP1000epnBGC. (C) TMC-86A **2** in extracts of *S. albus* containing pCAP1000tmcBGC. (D) Eponemycin congener **3** in extracts of *S.albus* containing pCAP1000epnBGC. (E) Eponemycin congener **4** in extracts of *S. albus* containing pCAP1000epnBGC. (F) Eponemycin congener **5** in extracts of *S. albus* containing pCAP1000epnBGC. (G) Eponemycin congener **6** in extracts of *S. albus* containing pCAP1000epnBGC. (H) Deshydroxy-eponemycin **11** in extracts of *S. albus* containing pCAP1000epnBGC-Δ*epnI*. (I) Deshydroxy-TMC-86A **12** in extracts of *S. albus* containing pCAP1000epnBGC-Δ*epnI*. (J) Deshydroxy-eponemycin congener 1**3** in extracts of *S. albus* containing pCAP1000epnBGC-Δ*epnI*. (K) Deshydroxy-eponemycin congener **14** in extracts of *S. albus* containing pCAP1000epnBGC-Δ*epnI*. (L) Deshydroxy-eponemycin congener **15** in extracts of *S. albus* containing pCAP1000epnBGC-Δ*epnI*. (M) Deshydroxy-eponemycin congener **16** in extracts of *S. albus* containing pCAP1000epnBGC-Δ*epnI*. (N) in extracts of *S. albus* containing pCAP1000epnBGC. (O) Dihydro-eponomycin **8** in extracts of *S. albus* containing pCAP1000epnBGC-Δ*epnJ.* (P) Dihydro-eponomycin congener **9** in extracts of *S. albus* containing pCAP1000epnBGC-Δ*epnJ*. (Q) Dihydro-TMC-86A **10** in extracts of *S. albus* containing pCAP1000epnBGC-Δ*epnJ*.

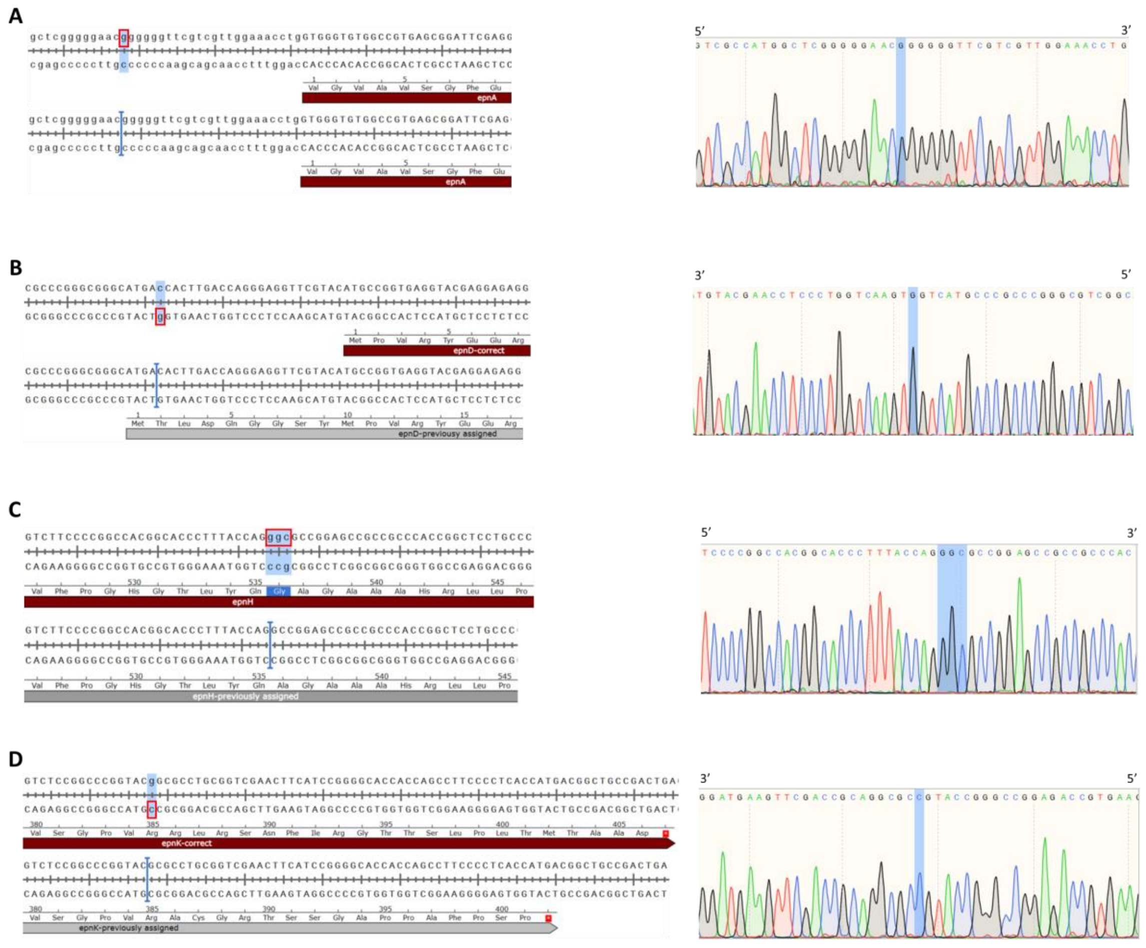

Fig. S4. Correction of errors in the originally reported sequence of the eponemycin BGC. The sequencing chromatograms for the relevant regions are shown on the right and the resulting changes to the sequence of the eponemycin BGC are shown on the left. (A) A guanine residue was missed 23 bp upstream of *epnA*. (B) A cytosine residue was missed in *epnD*, corresponding to a frame shift and a 27 bp extension at its 5' end. (C) A guanine-guanine-cytosine triplet was missed in *epnH*, corresponding to deletion of a glycine residue in EpnH. (D) A guanine residue was missed in *epnK*, corresponding to a 15 bp truncation at its 3' end.

Table S1. Bacterial strains and plasmids used in the study.

**Strain/Plasmid**	**Characteristics**	**Reference**
E. coli		
Top10	Host for general cloning	Invitrogen
ET12567	Donor strain for conjugal transfer from *E. coli* to *Streptomyces*	(1)
ET12567/pUB307	Helper strain for conjugal transfer from *E. coli* to *Streptomyces*	(1)
ET12567/pUZ8002	Donor strain for conjugal transfer from *E. coli* to *Streptomyces*	(1)
Streptomyces		
S. hygroscopicus ATCC53709	Eponemycin producer	(2)
S. chromofuscus ATCC49982	TMC-86A producer	(3)
S. albus J1074	Heterologous expression host	(4)
S. albus/epnBGC		This work
S. albus/tmcBGC-initial		This work
S. albus/tmcBGC		This work
S. albus/epnBGC-ΔepnD		This work
S. albus/epnBGC-ΔepnE		This work
S. albus/epnBGC-ΔepnDE		This work
S. albus/epnBGC-ΔepnI		This work
S. albus/epnBGC-ΔepnK		This work
S. albus/epnBGC-ΔepnJ		
S. albus/epnBGC-ΔepnI::epnI		This work
S. albus/epnBGC-ΔepnK::epnK		This work
S. albus/epnBGC-ΔepnJ::epnJ		
S. albus/tmcBGC-ΔtmcD		This work
S. albus/epnBGC-ΔepnD::*epnD*		This work
S. albus/epnBGC-ΔepnE::*epnE*		This work
S. albus/epnBGC-ΔepnDE::*epnDE*		This work
S. albus/tmcBGC-ΔtmcD::*tmcD*		This work
S. albus/tmcBGC-ΔtmcD::*epnD*		This work
S. albus/epnBGC::*epnDE*		This work

Saccharomyces		
S. cerevisiae VL6−48N		(5)
**Plasmid**		
pCR™2.1	Vector for sub-cloning and sequencing	Invitrogen
pCR-ADHURA3	pADH1-URA3 cloned on pCR™2.1 vector	This work
pCAP01		(6)
pCAP03		(7)
pCAP1000		This work
pCAP1000epn	pCAP1000-based vector for eponemycin BGC capture	This work
pCAP01epn	pCAP01-based vector for eponemycin BGC capture	This work
pCAP1000epnBGC	pCAP1000 derivative containing the eponemycin BGC	This work
pCAP1000tmc	pCAP1000-based vector for TMC-86A BGC capture	This work
pCAP01tmc	pCAP01-based vector for TMC-86A BGC capture	This work
pCAP03tmc	pCAP03-based vector for TMC-86A BGC capturie	This work
pCAP1000tmcBGC-initial	pCAP1000 derivative containing the eponemycin BGC	This work
pCAP1000tmcBGC	pCAP1000tmcBGC-initial derivative incorporating region upstream of *tmcA*	This work
pCAP1000epnBGC-ΔepnD	pCAP1000epnBGC derivative containing an in-frame deletion in *epnD*	This work
pCAP1000epnBGC-ΔepnE	pCAP1000epnBGC derivative containing an in-frame deletion in *epnE*	This work
pCAP1000epnBGC-ΔepnDE	pCAP1000epnBGC derivative containing an in-frame deletion in *epnDE*	This work
pCAP1000epnBGC-ΔepnI	pCAP1000epnBGC derivative containing an in-frame deletion in *epnI*	This work

pCAP1000epnBGC-ΔepnK	pCAP1000epnBGC derivative containing an in-frame deletion in *epnK*	This work
pCAP1000epnBGC-ΔepnJ	pCAP1000epnBGC derivative containing an in-frame deletion in *epnJ*	This work
pCAP1000tmcBGC-ΔtmcD	pCAP1000TMCBGC derivative containing an in-frame deletion in *tmcD*	This work
pOSV556t	Complementation vector	Kindly supplied by Prof. Jean-Luc Pernodet, Université Paris-Sud 11
pOSV-epnD	Δ*epnD* complementation construct	This work
pOSV-epnE	Δ*epnE* complementation construct	This work
pOSV-epnDE	Δ*epnDE* complementation construct	This work
pOSV-tmcD	Δ*tmcD* complementation construct	This work
pOSV-epnI	Δ*epnI* complementation construct	This work
pOSV-epnK	Δ*epnK* complementation construct	This work
pOSV-epnJ	Δ*epnJ* complementation construct	This work

Table S2. Oligonucleotide primers used in the study.

Primer	Oligonucleotide sequences (5' to 3')	
ADH1-F	agatcgcctaggcctacaacaactaagaaaatggctat catgcggaagctggtgagaag	*pADH1* of pCR-ADHURA3
ADH1-R	ctttcgacatcatatggtttaaacggatccccactattta taccatgggaggcgt	*pADH1* of pCR-ADHURA3
URA3-F	taaatagtggggatccgtttaaaccatatgatgtcgaa agctacatataaggaac	*URA3* of pCR-ADHURA3
URA3-R	tgtattcctaggttagttttgctggccgcatcttctcaaat atgcttccc	*URA3* of pCR-ADHURA3
ADH1-URA3-final-F	agatcgactagtcctacaacaactaag	pCAP1000 counter-selectable cassette
ADH1-URA3-final-R	tcaagtctcgagttagttttgctggc	pCAP1000 counter-selectable cassette
pCAP1000-CK1-F	ctttcagcttcgatgtagg	pCAP1000 checking primer 1
pCAP1000-CK1-R	tctttgtctttgttcttcc	pCAP1000 checking primer 1
pCAP1000-CK2-F	gatgctaaggtagagggtg	pCAP1000 checking primer 2
pCAP1000-CK2-F	tcggggaaatgtgcg	pCAP1000 checking primer 2
1000-epn-L-F	caaagatcgactagtatacaaaggtcacttaccc	pCAP1000epn left capture arm
1000-epn-L-R	cttagttgttgtaggtcccggaatactgtg	pCAP1000epn left capture arm
1000-epn-M-F	cacagtattccgggacctacaacaactaag	pCAP1000epn counter-selectable cassette
1000-epn-M-R	agtgccttctgcgccttagttttgctggccgcat	pCAP1000epn counter-selectable cassette
1000-epn-R-F	ggccagcaaaactaaggcgcagaaggcacttctc	pCAP1000epn right capture arm
1000-epn-R-F	acctcaagtctcgagtcaggagtggaggaa	pCAP1000epn right capture arm
01-epn-L-R	ctgcgccgtttaaactcccggaatactgtggtatgtcac a	pCAP01epn left capture arm
01-epn-R-F	tccgggagtttaaacggcgcagaaggcacttctccagg cc	pCAP01epn right capture arm
epnBGC-CK1-F	tgccgtcatcaggggacc	epnBGC PCR checking primer 1
epnBGC-CK1-R	ccccgccgatgagcagtt	epnBGC PCR checking primer 1
epnBGC-CK2-F	cctccgatttgaagggtgatg	epnBGC PCR checking primer 2
epnBGC-CK2-R	gccagggtgaagtcgtccag	epnBGC PCR checking primer 2
epnBGC-CK3-F	gcctcaatgatgacttcttcgc	epnBGC PCR checking primer 3
epnBGC-CK3-R	cgtccttcgggttcttcctg	epnBGC PCR checking primer 3
1000-TMC-L-F	ttctacaaagatcgactagtcggcaggagtcgaccatg c	pCAP1000tmc left capture arm

1000-TMC-L-R	ttgttgtaggcctaggctgaccatgtcggacgcc	pCAP1000tmc left capture arm
1000-TMC-M-F	catggtcagcctcctaggacaacaactaagaaaa	pCAP1000tmc counter-selectable marker
1000-TMC-M-R	gcggccgccgcctaggttagttttgctggccgcat	pCAP1000tmc counter-selectable marker
1000-TMC-R-F	gcaaaactaacctaggcggcggccgcgcgcacg	pCAP1000tmc right capture arm
1000-TMC-R-R	acaggtacctcaagtctcgagttttgagggaggtcctg gt	pCAP1000tmc right capture arm
01-TMC-L-R	ccgccggtttaaacaccggtctgaccatgtcggacgcc ttggagt	pCAP01tmc left capture arm
01-TMC-R-F	tggtcagaccggtgtttaaaccggcggccgcgcgcacg ccccgga	pCAP01tmc right capture arm
TMCBGC-CK1-F	gcgaagcgggcgatgagacc	tmcBGC PCR checking primer 1
TMCBGC-CK1-R	gccgtccatcaccgaactgc	tmcBGC PCR checking primer 1
TMCBGC-CK2-F	ttctccttaccggtgagaagatc	tmcBGC PCR checking primer 2
TMCBGC-CK2-R	gtaatacgtgaactggctcatgc	tmcBGC PCR checking primer 2
TMCBGC-CK3-F	gtgtttccgttcatgtccgc	tmcBGC PCR checking primer 3
TMCBGC-CK3-R	gcccttgtcctcgtgcctc	tmcBGC PCR checking primer 3
tmcA-upstreamregion-F	tttcgcggtggacagaccgcacgccacaccctcaagaa tcctgcggcccatgtcgctcacagtgaccttctt	*tmcA* upstream region
tmcA-upstreamregion-R	gtgcgcggaacccctatttgtttatttttctaaatacagg tacctcaagtctcgagcggttcgtcggaggggccgcgc gca	*tmcA* upstream region
TMCBGC-P-CK-F	cagcagacccaccgagtag	pCAP1000tmcBGC promoter fixing checking primer
TMCBGC-P-CK-R	gagcaggaagagcaagataaaa	pCAP1000tmcBGC promoter fixing checking primer
epnD-F	ggcaagctttggaattctgccgacgcccgggcgggcat gaccacttgacc	*epnD* for complementation
epnD-R	gtaaggccttcaggcagtgggcagcttgggccaagtga gcgtgg	*epnD* for complementation
epnE-F	ggcaagcttcacccccctacacagaggaatgggacgtc ccatgttcgagacgc	*epnE* for complementation
epnE-R	gtaaggccttcaggccgccgcgttcctttcctctatcag cttcg	*epnE* for complementation
tmcD-F	ggcaagcttcgaaggaggttgtgcatgccggtgaggc acgagga	*tmcD* for complementation
tmcD-R	gagctcgagtcaggcggtggggagcgccggccaggtg ag	*tmcD* for complementation
tmcE-F	ggcaagcttccacaaaggaatctgccccccatgttcga gacgctcaaaga	*tmcE* for complementation
tmcE-R	gtactcgagtcagagcgccgcgttcctctcctccatcag	*tmcE* for complementation
epnI-F	accggcatgcaagcttatcgcagaaagagaatcactg atg	*epnI* for complementation
epnI-R	atgcaagctatcaggccttcaggcatggacgttctctcc tgc	*epnI* for complementation

epnK-F	accggcatgcaagcttcgagaaggaggacgatctcgt atgac	*epnK* for complementation
epnK-R	gcatgcaagctatcaggccttcagtcggcagccgtc	*epnK* for complementation
pOSV556t-CK-F	taagggacagtgaagaaggaa	pOSV556t sequencing primer
pOSV556t-CK-R	caagcataaagctggcatg	pOSV556t sequencing primer
RP-1-F	cagggcgaagaatctcgtgcttt	Repair polynucleotides 1 in *epnI* and *epnK* deletion
RP-1-R	cgatcttgagcaggcccatcgaata	Repair polynucleotides 1 in *epnI* and *epnK* deletion
RP-2-F	ggggagcgttgaagaaactctaccg	Repair polynucleotides 2 in *epnI* and *epnK* deletion
RP-2-R	gctgtggaacggtccttcggg	Repair polynucleotides 2 in *epnI* and *epnK* deletion
RP-3-F	tggaccgccagctcaaggtg	Repair polynucleotides 3 in *epnI* and *epnK* deletion
RP-3-R	ttccggcgtagaccccgatccag	Repair polynucleotides 3 in *epnI* and *epnK* deletion
RP-4-F	actaccgctcgcccaaccc	Repair polynucleotides 4 in *epnI* and *epnK* deletion
RP-4-R	gtgagctgtccgggtttcaccgg	Repair polynucleotides 4 in *epnI* and *epnK* deletion
RP-5-F	tgatgatcttcgccgggcacgagac	Repair polynucleotides 5 in *epnI* and *epnK* deletion
RP-5-R	gggcagcagataggcgatgtggtc	Repair polynucleotides 5 in *epnI* and *epnK* deletion
RP-6-F	gagacggcgtacaacgatcacac	Repair polynucleotides 6 in *epnI* and *epnK* deletion
RP-6-R	tcggatgagaaatcagacgccgc	Repair polynucleotides 6 in *epnI* and *epnK* deletion
epnI-DP-L-F	ccagcttcctcctcggcggcctggtctac	*epnI* upstream regions for *epnI* deletion polynucleotides
epnI-DP-L-R	ccgacagggacggaaaggggtcggcgttgaacgta	*epnI* upstream regions for *epnI* deletion polynucleotides
epnI-DP-R-F	cccctttccgtccctgtcggcggacgtgccggca	*epnI* downstream regions for *epnI* deletion polynucleotides
epnI-DP-R-R	ccagcggcacatacgcggccccggtcttgaggac	*epnI* downstream regions for *epnI* deletion polynucleotides
epnK-DP-L-F	gccgacctgcgggaagcggccgcgtcgtcgat	*epnK* upstream regions for *epnK* deletion polynucleotides
epnK-DP-L-R	aggcgccgtacgaagatcatcggggcgtc	*epnK* upstream regions for *epnK* deletion polynucleotides
epnK-DP-R-F	gatgatcttcgtacggcgcctgcggtcgaacttcat	*epnK* downstream regions for *epnK* deletion polynucleotides
epnK-DP-R-R	ccagcggcacatacgcggccccggtcttgaggac	*epnK* downstream regions for *epnK* deletion polynucleotides
epnI-CK-F	ctggtctacaagacccaggc	*epnI* deletion checking primer
epnI-CK-R	gggcagcagataggcgat	*epnI* deletion checking primer
epnK-CK-F	ccaccatgtgcgtgatgt	*epnK* deletion checking primer

epnK-CK-R	aggtgctcactgctccaac	*epnK* deletion checking primer
RP-7-F	agcaggagagaacgtccatgcctga	Repair polynucleotides 7 in *epnD* deletion and *epnDE* deletion
RP-7-R	gcccagagcgaagaagtcatcattgag	Repair polynucleotides 7 in *epnD* deletion and *epnDE* deletion
epnJ-DP-L-F	tgatcttcgccgggcacgagaccacggtgcagttgatc at	*epnJ* upstream region for *epnJ* deletion polynucleotides
epnJ-DP-L-R	ggttttggagcggacctcgaccagcgagtggttcgcgg ag	*epnJ* upstream region for *epnJ* deletion polynucleotides
epnJ-DP-R-F	cactcgctggtcgaggtccgctccaaaaccggtctcgt gct	*epnJ* downstream region for *epnJ* deletion polynucleotides
epnJ-DP-R-R	gagctcatgggcgtcggtgtgggagacgcccatcatgg tc	*epnJ* downstream region for *epnJ* deletion polynucleotides
RP-8-F	gacccactgtcgccctgtgaacc	Repair polynucleotides 8 in *epnJ* deletion
RP-8-R	ttctttgagcgtctcgaacatgg	Repair polynucleotides 8 in *epnJ* deletion
epnJ-CK-F	ggtgaccatccccaagggctcgt	*epnJ* deletion checking primer
epnJ-CK-R	catcccattgctgaaggcacggt	*epnJ* deletion checking primer
epnD-DP-L-F	gaggaaggctacgagcggatggacgag	*epnD* upstream region for *epnD* deletion polynucleotides
epnD-DP-L-R	tgggcagcttctcctcgtacctcaccggcatgtac	*epnD* upstream region for *epnD* deletion polynucleotides
epnD-DP-R-F	gtacgaggagaagctgcccactgcctgacaccccc	*epnD* downstream region for *epnD* deletion polynucleotides
epnD-DP-R-R	gcgttcctgcgtgggaacacgtttc	*epnD* downstream region for *epnD* deletion polynucleotides
DE-DR-R-F	gtacgaggaggaaaggaacgcggcggcctgac	*epnE* downstream region for *epnDE* deletion polynucleotides
DE-DR-R-R	taccagcgggaaagggggggaacggc	*epnE* downstream region for *epnDE* deletion polynucleotides
DE-DR-L-R	cgttcctttcctcctcgtacctcaccggcat	*epnD* upstream region for *epnDE* deletion polynucleotides
epnD-CK-F	ctgtgaaccggcagcctc	*epnD* deletion checking primer
epnD-CK-R	cgtgatcgtgaaatccttc	*epnD* deletion checking primer
epnDE-CK-F	ccctgccgtaacgactcgccatg	*epnDE* double deletion checking primer
epnDE-CK-R	cccgtctttccggtgaacgtgcc	*epnDE* double deletion checking primer
RP-9-R	cccgactacgtggtcctgctcat	Repair polynucleotides 9 in *epnE* deletion
RP-9-R	ccagttccttcgccagcagtgtc	Repair polynucleotides 9 in *epnE* deletion
epnE-DP-L-F	gcaggttcgcacacctcatccaggt	*epnE* upstream region for *epnE* deletion polynucleotides
epnE-DP-L-R	cgttcctttctacttctttgagcgtctcgaa	*epnE* upstream region for *epnE* deletion polynucleotides

epnE-DP-R-F	caaagaagtagaaaggaacgcggcggcctgac	*epnE* downstream region for *epnE* deletion polynucleotides
epnE-DP-R-R	taccagcgggaaagggggggaacggc	*epnE* downstream region for *epnE* deletion polynucleotides
epnE-CK-F	gagggacgtgacacgttccggcagg	*epnE* deletion checking primer
epnE-CK-R	gcatcgggcgacggcgatgagac	*epnE* deletion checking primer
tmcD-DP-L-F	atgccggtgaggcactggccggcgctccccaccgc	*tmcD* upstream region for *tmcD* deletion polynucleotides
tmcD-DP-L-R	tcggtgatcgcgatcgcgtggtccttc	*tmcD* upstream region for *tmcD* deletion polynucleotides
tmcD-DP-R-F	ggggagcgccggccagtgcctcaccggcatgcacaac	*tmcD* downstream region for *tmcD* deletion polynucleotides
tmcD-DP-R-R	ttcgtcggcaaggcttcccaggattg	*tmcD* downstream region for *tmcD* deletion polynucleotides
RP-10-F	gaataagggacagtgaagaagg	Repair polynucleotides 10 in *tmcD* deletion
RP-10-R	agagaactcaaaggttaccc	Repair polynucleotides 10 in *tmcD* deletion
RP-11-F	atggccaaccgggtcgccgactc	Repair polynucleotides 11 in *tmcD* deletion
RP-11-R	tcccggccggcgcgatcatg	Repair polynucleotides 11 in *tmcD* deletion
RP-12-F	gtggtagtcgacgttgtcccggt	Repair polynucleotides 12 in *tmcD* deletion
RP-12-R	cgtcctgcggcccggaca	Repair polynucleotides 12 in *tmcD* deletion
RP-13-F	agcgcgtcctccaggcggcgca	Repair polynucleotides 13 in *tmcD* deletion
RP-13-R	acggtacgccggctcgcccgct	Repair polynucleotides 13 in *tmcD* deletion
tmcD -CK-F	ctcctccatcagtctcgc	*tmcD* deletion checking primer
tmcD -CK-R	cgaactcgtcggaaatct	*tmcD* deletion checking primer

Primer pairs for amplification of left- or right-homologous arms and counter-selectable markers, for PCR/sequencing-based verification, and for complementation plasmid construction are identified by the suffixes -L-F/-L-R, -R-F/-R-R, -M-F/-M-R, -F/-R, and CK-F/CK-R, respectively.

Table S3. Comparison of the efficiency of eponemycin and TMC-86A BGC capture using pCAP01, pCAP03 and pCAP1000-derived vectors. Less than 50 colonies in total are obtained in a single experiment.

	Captured size	Digested DNA fragments	pCAP03 (Correct/Checked)	pCAP01 (Correct/Checked)	pCAP1000 (Correct/Checked)
**epnBGC**	**23kb**	**>24kb**	**0/0**	**7/30**	**4/6**
**tmcBGC**	**28kb**	**~32kb**	**0/0**	**0/13**	**4/4**

Table S4. Summary of modifications made to the eponemycin and TMC-86A BGCs following TAR capture. More than 100 colonies are obtained in a typical experiment.

Mutants	Restriction enzyme	No. of Restriction sites	DNA fragments (DP + RP)	Length of Homologous flanking regions	Screening (correct/checked)
tmcBGC	*XhoI*	1	*tmcA* upstream region	~50bp	5/8
△epnK	*Eco*RI	8	epnK-DP + RP 2, 3, 4, 5, 6	~500bp	2/13
△epnI			epnI-DP + RP 1, 3, 4, 5, 6	~500bp	3/15
△epnJ	*SrfI*	4	epnJ-DP + RP 8	~500bp	7/8
△epnD			epnD-DP + RP 7	~500bp	2/3
△epnDE			epnDE-DP + RP 7	~500bp	3/3
△epnE	*ScaI*	2	epnE-DP + RP 9	~500bp	3/3
△tmcD	*SphI*	8	tmcD-DP + RP 10, 11, 12, 13, 14*	~230bp - 850bp	1/14

*RP14 is the amplimer from pCAP1000tmcBGC using TMCBGC-P-CK-F and TMCBGC-P-CK-R primers.

References

1. MacNeil,D.J., Gewain,K.M., Ruby,C.L., Dezeny,G., Gibbons,P.H. and MacNeil,T. (1992) Analysis of *Streptomyces avermitilis* genes required for avermectin biosynthesis utilizing a novel integration vector. Gene., 111, 61–68.
2. Schorn,M., Zettler,J., Noel,J.P., Dorrestein,P.C., Moore,B.S. and Kaysser,L. (2014) Genetic basis for the biosynthesis of the pharmaceutically important class of epoxyketone proteasome inhibitors. ACS Chem Biol., 9, 301–309.
3. Zabala,D., Cartwright,J.W., Roberts,D.M., Law,B.J.C., Song,L., Samborskyy,M., Leadlay,P.F., Micklefield,J. and Challis,G.L. (2016) A flavin-dependent decarboxylase-dehydrogenase-monooxygenase assembles the warhead of α,β-epoxyketone proteasome inhibitors. J Am Chem Soc., 138, 4342–4345.
4. Lombó,F., Velasco,A., Castro,A., de la Calle,F., Braña,A.F., Sánchez-Puelles,J.M., Méndez,C. and Salas,J.A. (2006) Deciphering the biosynthesis pathway of the antitumor thiocoraline from a marine actinomycete and its expression in two *Streptomyces* species. ChemBioChem., 7, 366–376.
5. Noskov,V.N., Kouprina,N., Leem,S.-H., Ouspenski,I., Barrett,J.C. and Larionov,V. (2003) A general cloning system to selectively isolate any eukaryotic or prokaryotic genomic region in yeast. BMC Genomics., 4, 16.
6. Yamanaka,K., Reynolds,K.A., Kersten,R.D., Ryan,K.S., Gonzalez,D.J., Nizet,V., Dorrestein,P.C. and Moore,B.S. (2014) Direct cloning and refactoring of a silent lipopeptide biosynthetic gene cluster yields the antibiotic taromycin A. Proc Natl Acad Sci U S A., 111, 1957–1962.
7. Tang,X., Li,J., Millán-Aguiñaga,N., Zhang,J.J., O’Neill,E.C., Ugalde,J.A., Jensen,P.R., Mantovani,S.M. and Moore,B.S. (2015) Identification of thiotetronic acid antibiotic biosynthetic pathways by target-directed genome mining. ACS Chem Biol., 10, 2841–2849.
